# Metabotropic glutamate receptor 5 knockout rescues obesity phenotype in a mouse model of Huntington’s disease

**DOI:** 10.1038/s41598-022-08924-4

**Published:** 2022-04-04

**Authors:** Rebeca P. M. Santos, Roberta Ribeiro, Talita H. Ferreira-Vieira, Rosaria D. Aires, Jessica M. de Souza, Bruna S. Oliveira, Anna Luiza D. Lima, Antônio Carlos P. de Oliveira, Helton J. Reis, Aline S. de Miranda, Erica M. L. Vieira, Fabiola M. Ribeiro, Luciene B. Vieira

**Affiliations:** 1 Departamento de Farmacologia, ICB, Universidade Federal de Minas Gerais, Ave. Antonio Carlos 6627, Belo Horizonte, MG CEP 31270-901 Brazil; 2 Departamento de Morfologia, ICB, Universidade Federal de Minas Gerais, Belo Horizonte, Brazil; 3 Departamento de Bioquímica e Imunologia, ICB, Universidade Federal de Minas Gerais, Belo Horizonte, CEP 31270-901 Brazil; 4 Faculdade Sete Lagoas, Sete Lagoas, Brazil

**Keywords:** Feeding behaviour, Neuroimmunology, Metabolism

## Abstract

Obesity represents a global health problem and is characterized by metabolic dysfunctions and a low-grade chronic inflammatory state, which can increase the risk of comorbidities, such as atherosclerosis, diabetes and insulin resistance. Here we tested the hypothesis that the genetic deletion of metabotropic glutamate receptor 5 (mGluR5) may rescue metabolic and inflammatory features present in BACHD mice, a mouse model of Huntington’s disease (HD) with an obese phenotype. For that, we crossed BACHD and mGluR5 knockout mice (mGluR5^−/−^) in order to obtain the following groups: Wild type (WT), mGluR5^−/−^, BACHD and BACHD/mGluR5^−/−^ (double mutant mice). Our results showed that the double mutant mice present decreased body weight as compared to BACHD mice in all tested ages and reduced visceral adiposity as compared to BACHD at 6 months of age. Additionally, 12-month-old double mutant mice present increased adipose tissue levels of adiponectin, decreased leptin levels, and increased IL-10/TNF ratio as compared to BACHD mice. Taken together, our preliminary data propose that the absence of mGluR5 reduce weight gain and visceral adiposity in BACHD mice, along with a decrease in the inflammatory state in the visceral adipose tissue (VAT), which may indicate that mGluR5 may play a role in adiposity modulation.

## Introduction

Obesity is a chronic and multifactorial disease characterized by an excess of body adiposity, which affects homeostasis through the disruption in peripheral and central mechanisms associated with the control of energy balance^1^. Moreover, excess of fat stored in the adipose tissue can lead to hypertrophy, hyperplasia, hypoxia, and consequently adipocyte necrosis^2,3^. In addition, insulin resistance, increased and decreased leptin and adiponectin levels, as well as recruitment of macrophages, T lymphocytes, and production of IL-1β, IL-4, IL-6, IL-17, and TNF-α, lead to local and systemic chronic low-grade inflammation^4–6^. Furthermore, obesity also impairs the function of an important central region in the control of energy balance, the hypothalamus, through mechanisms including neuroinflammation, deficits in neurotransmission, neurodegeneration, and alterations in the expression of orexigen and anorexigen neuronal populations^7–11^.

The modulation of metabolic, inflammatory, and energy balance pathways may be an interesting therapeutic strategy for obesity and its potential commorbidities^12^. Recent data point out that the deletion or the antagonism of mGluR5, a subtype of the group I mGluR5 coupled to phospholipase C through Gq protein^13^, is associated with the regulation of metabolic parameters in mice fed with high fat diet^14,15^.

mGluR5 is widely expressed in central nervous system (CNS) regions associated with body homeostasis, consumption and energy expenditure, such as hypothalamus. Furthermore, it is found in the cells of innate and adaptive immunity, including macrophages, lymphocytes B and T^16–18^. Notably, some studies suggest that this receptor may contribute to fat deposition, metabolic dysfunction, and may also cause a systemic low grade chronic inflammatory state, especially in obesity^14,15,19^.

Huntington disease (HD) is an autosomal dominant neurodegenerative disorder, in which symptoms are motor alterations, cognitive dysfunction, and psychiatric disorders. HD is caused by an expansion of CAG sequence in the *Huntingtin* gene (IT-15), which leads to an altered huntingtin protein (HTT) that possesses an elongated polyglutamine tract^20,21^. HD patients suffers for an involuntary weight loss, nonetheless it is unknown how the extent weight loss influences disease progression in HD^22^. In this context, pre-clinical data suggest that the deregulation of energy expenditure and body weight may offer gradual catalysts for the neurodegeneration^20,21,23–26^. Interestingly in the early stage of HD, the aggregation of HTT in the hypothalamus and adipose tissue is related with hyperphagia, insulin resistance, alteration in levels and function of adipokines, such as leptin, and abnormal metabolism and fat storage by adipocytes, which may be associated with endocrine abnormalities, peripheral/central inflammation, and weight gain^12,22,23,25,27–31^. Furthermore, BACHD, a mouse model of HD which expresses full-length human HTT, exhibiting progressive neurodegeneration, alongside with increased body weight, impaired glucose metabolism and pronounced insulin and leptin resistance^27^. Importantly, other mice models expressing full-length HTT present increased body weight as BACHD mice^30,32–34^. Although obesity is not a symptomatic feature of HD, metabolic and obese phenotype of HD mice models which expresses full-length human HTT, such as BACHD, may offer an interesting tool for studying metabolic alterations associated with obese environment, especially in a context of late neurodegeneration. Thus, in order to understand the role of mGluR5 in obesity, besides how its ablation modulates obese features in BACHD mice, we designed the present study to test whether the absence of mGluR5 signaling improves metabolic and inflammatory dysfunctions associated with BACHD mice^27^. Importantly, our preliminary findings indicate that deletion of mGluR5 might be associated with a reduction of body weight and modulation of adipose tissue, revealing a strong improvement in the peripheral effects related to the obesity phenotype observed in BACHD mice.

## Experimental procedures

### Animals

**Figure 1:**
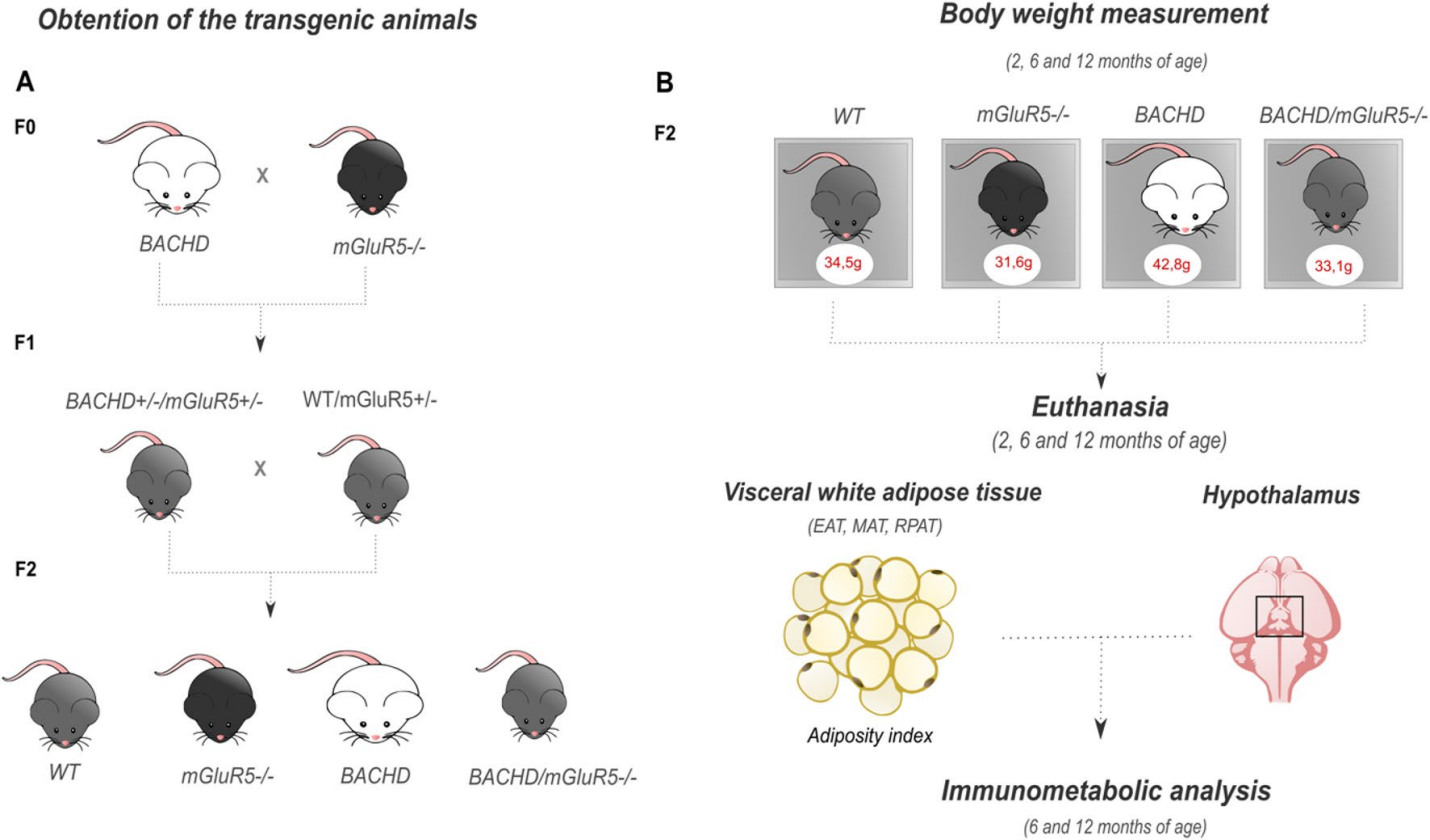
Schematic representation of experimental design. (**A**) Generation of the mice lines. FVB/NJ, FVB/N-Tg (HTT*97Q) IXwy/J (BACHD) and mGluR5 KO mice (mGluR5^−/−^) mice were crossed over to generate the double mutant mice (BACHD/mGluR5^−/−^ mice) (F1). The F2 generation containing all genotypes used in this study was obtained by crossing between F1 generation. **(B**) From 2 to 12 months of age, mice were weighted once a month and adiposity index was measured at 2, 6 and 12 months in all tested genotypes. Subsequently at 2, 6 and 12 months of age mice were euthanized for obtaining visceral white adipose tissues and the hypothalamus in order to perform immunologic and metabolic analysis. *EAT* epidydimal adipose tissue, *MAT* mesenteric adipose tissue, *RPAT* retroperitoneal adipose tissue.

FVB/NJ (WT mice), FVB/N-Tg (HTT*97Q) IXwy/J (BACHD)), and mGluR5 KO mice (C57/B6; 129-Grm5tm1Rod/J (mGluR5^−/−^)) were purchased from the Jackson laboratory (Bar Harbor, USA). We used four lineages in our study, including wild type (WT), mGluR5^−/−^, BACHD, and the double mutant mice (BACHD/mGluR5^−/−^). For obtaining all genotypes, we performed the crossing in accordance to previous literature^35^. In summary, the first generation (F1) was originated by crossing over BACHD and mGluR5^−/−^. Subsequently, we crossed over the F1 generation, resulting in the littermates (F2) with interesting genotype (Fig. 1A). In addition, genotyping was performed using polymerase chain reaction (PCR) (Supplementary data). Due to low fertility of mGluR5 KO mice, only homozygous males between 2, 6, and 12 months of age were used in the following experiments. Mice were housed in an animal facility care at 23 °C, on a light/darker cycle of 12 h with water and food ad libitum. In addition, euthanasia was performed by intraperitoneal anesthesia with ketamine/xylazine (80/8 mg/kg). Housing and experimentations were carried out in compliance with the ARRIVE guidelines and according to out in accordance with the guidelines of the Brazilian National Council of the Control of Animal Experimentation (CONCEA) and approved by the Ethics Committee on Animal Use of Federal University of Minas Gerais under the protocol number 234/2016.

### Body weight and visceral adipose tissue index

Mice from 2 to 12 month-old were weighed once a month. In order to quantify the adiposity, at 2, 6 and 12 months, epididymal (EAT), mesenteric (MAT), and retroperitoneal (RPAT) fat pad were collected, weighed and used for the calculation of visceral adipose tissue index, obtained by the sum of the weight of all visceral adipose tissue fat pads (VAT), normalized by body weight of individual mice (Fig. 1B).

### Measurement of adipokines and cytokines

Mice were euthanized at 6 and 12 months of age, the hypothalamus and visceral adipose tissues (EAT, MAT, and RPAT) were collected and homogenized in an extraction solution (100 mg of tissue per milliliter), containing Tris–HCl (20 mM); NaCl (137 mM); NP40 (1%); Glycerol (10%); phenyl methyl sulfonylfluoride (1 mM) or aprotinin A (0,5 µg/mL), Pesptatin A (1 μM), EDTA (10 mM), E-64 (10 μM), sodium vanadate (0,5 mM), and deionized water. Lysates were centrifuged (13,000 g, 4 °C, 20 min), and supernatant was obtained and stocked at − 80 °C until use. The levels of the adipokines, leptin and adiponectin, were measured using a commercial kit (Mouse-Leptin and Adiponectin/Acrp30 Duoset ELISA—R&D Systems, Minneapolis, MN)) in accordance to manufactory instructions. The concentration of cytokines IL-2, IL-4, IL-6, IL-10, TNF, IFN-γ and IL-17A was determined using a CBA commercial kit (mouse Th1/Th2/Th17-BD Biosciences, San Diego, CA). The results were acquired on FACS CANTO II flow cytometer (Becton Dickison, San Jose, USA), and analyzed in the software FCAP array (Soft Flow Inc. Pecs, Hungary).

### Statistical analysis

Statistical analyses were performed using the software GraphPad Prism 7 (San diego, USA). We employed the software G Power 3.1 (Dusseldorf, Germany) to calculate the sample size and power of the statistical analysis.The normality and homoscedasticity of the data was testing using Shapiro–wilk and levene’s test, respectively. Before statistical tests, all data were analyzed by ROUT method for outlier detection, and extreme values were excluded from the analysis^36^. Results were expressed as mean ± SEM, and the comparison between multiple groups was performed using one-way analysis of variance (ANOVA) following by Bonferroni post hoc test. Furthermore, repeated measures ANOVA, and mixed effect model, followed by Tukey post hoc test were used in the analysis of repeated measures data. In addition, for non homogeneity parametric data, and for non parametric data, One-Way Welch ANOVA followed by Games-Howell’s post hoc test, and Kruskal Wallis followed by Dunn’s post hoc test were respectively performed as indicated in the figure legends. Statistical significance was defined by p < 0.05.

## Results

### Genetic deletion of mGluR5 prevent increased body weight in BACHD mice

**Figure 2:**
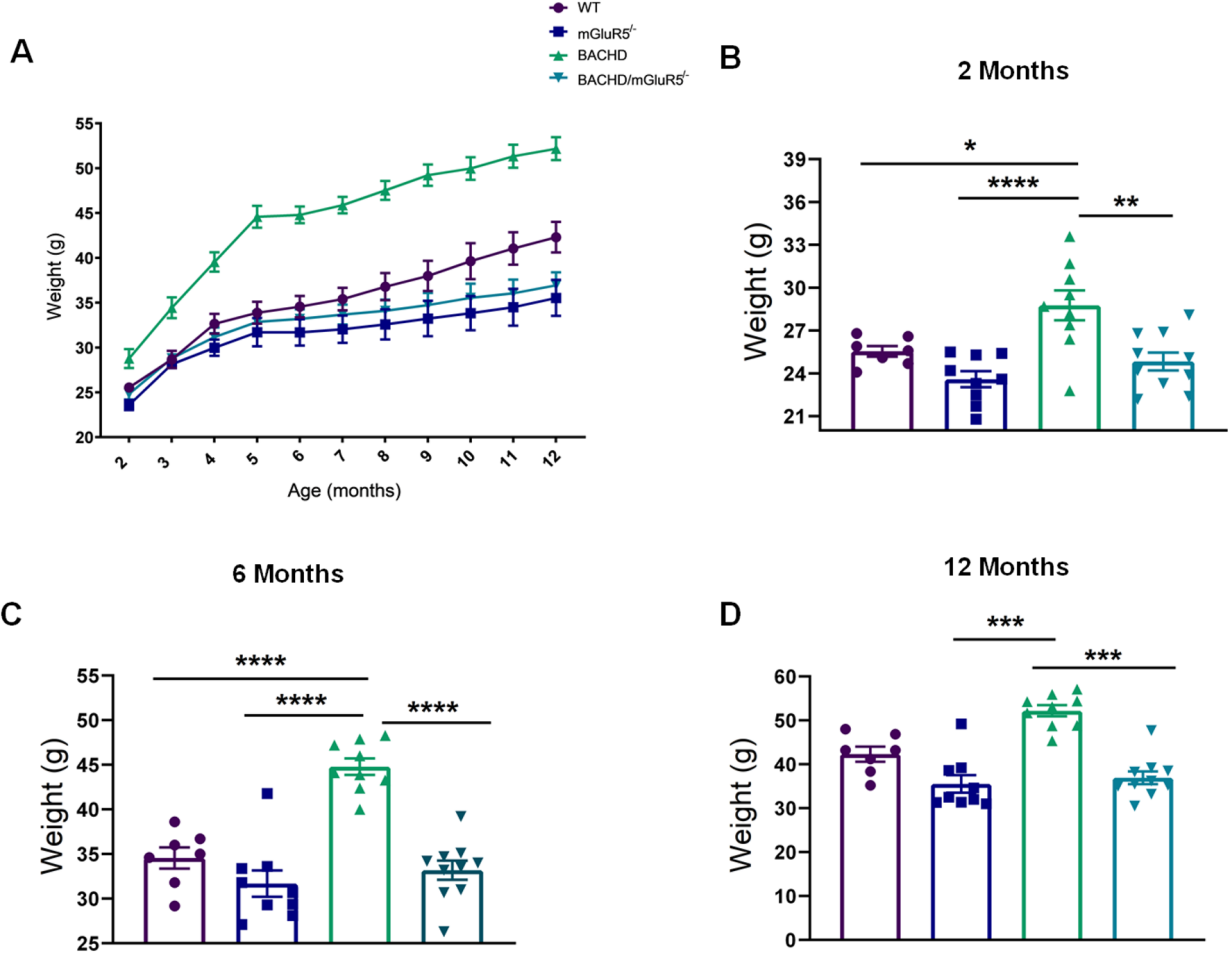
mGluR5 genetic deletion decreases body weight in BACHD mice. (**A**) Body weight measurements of WT, mGluR5^−/−^, BACHD and BACHD/mGluR5^−/−^ mice from 2 to 12 months showed an interaction effect between time and genotype (F(_30,329_) = 4,302). (**B**–**D**) Body weight of all tested groups at 2, 6 and 12 months of age. Error bars represent the mean ± SEM; n = 7–11. Repeated measures ANOVA and mixed model followed by Tukey post test (**A**). One-Way ANOVA followed by Bonferroni (**B**–**C**), and Kruskal–Wallis followed by Dunn’s post test (**D**). *p < 0.05; **p < 0.01; ***p < 0.001; ****p < 0.0001.

**Figure 3:**
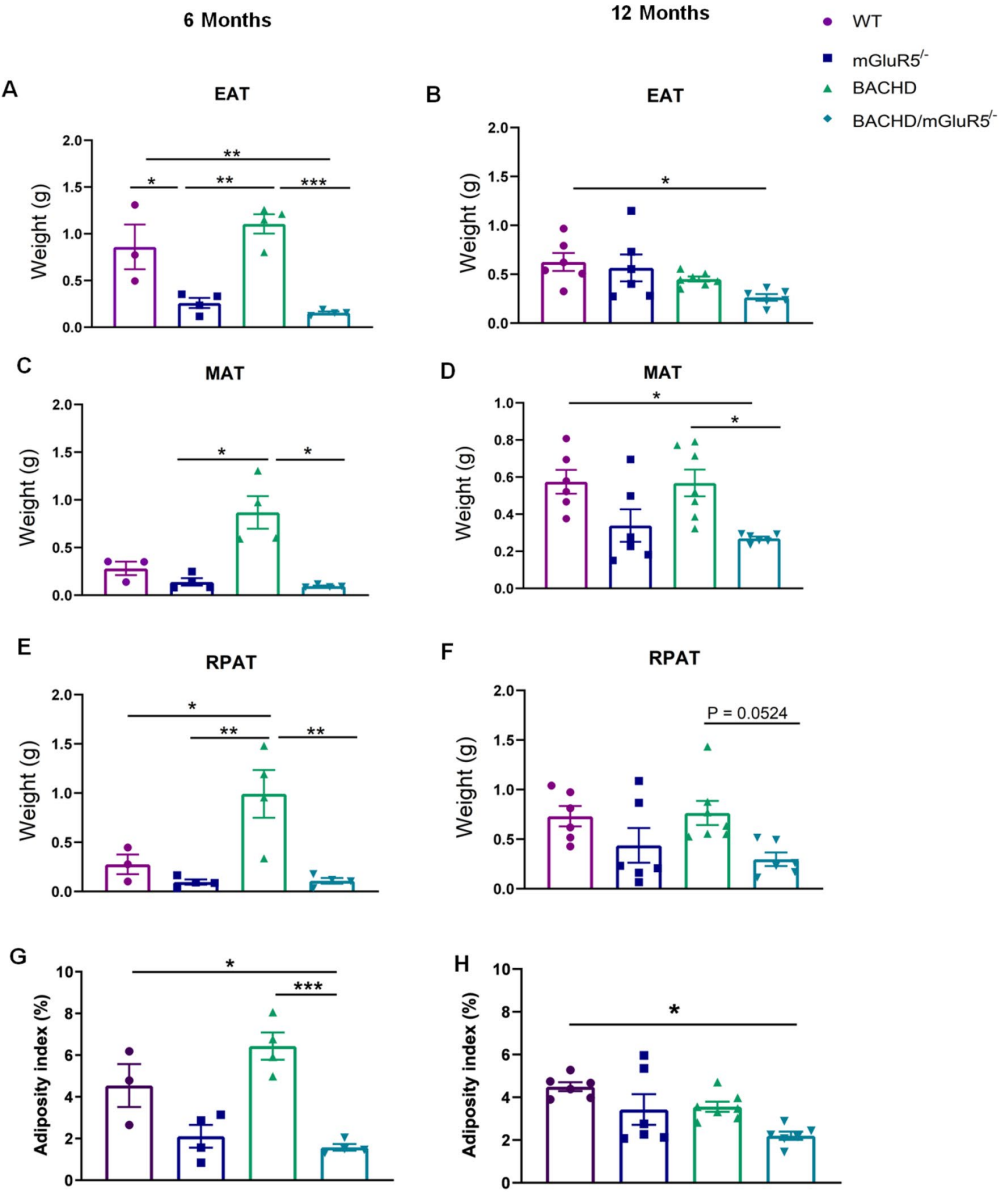
mGluR5 genetic deletion reduces fat pads wet weight and adiposity indices in BACHD mice at 6 months of age. All fat pads were individually weighted, and the adiposity index was obtained by the sum of the weight of visceral adipose tissue fat pads (VAT), normalized by body weight of individual mice. (**A**–**F**) measurement of the wet weight of the epididymal (EAT), mesenteric (MAT), retroperitoneal (RPAT) adipose tissue of WT, mGluR5, BACHD and BACHD/mGluR5^−/−^ mice, at 6 and 12 months of age, left and right columns, respectively. (**G, H)** Adiposity index of WT, mGluR5^−/−^, BACHD and BACHD/mGluR5^−/−^ mice from 6 and 12 months of age. Error bars represent the mean ± SEM; n = 3–8. One-Way ANOVA followed by Bonferroni post test (**A**, **B**, **D**, **E,** **G**), and Krustal-Wallis followed by Dunn’s post test (**C**, **F**, **H**). *p < 0.05, **p < 0.01, ***p < 0.001; ****p < 0.0001.

First, we decided to investigate the effect of mGluR5 deletion on body weight and visceral fat in BACHD mice (Figs. 2, 3). Figure 2A shows body weight measurements of all mice lines from 2 to 12 months of age. Figure 2B–D display graphical analyzes of the body weight at 2, 6 and 12 months of all tested groups. Results showed that an interactive effect between time and genotype is associated with the increase of body weight in all lineages of interesting (Repeated measures ANOVA and mixed model, F(_30,329_) = 4,302, p < 0.0001) (Fig. 2A). In this context, at 2 and 6 months of age, BACHD mice present the highest body weight as compared to all genotypes (Fig. 2B–D). However, at 12 months of age, our statistical analyses did not detect a body weight difference as compared to BACHD mice and WT mice (Kruskal–Wallis, p = 0.2463) (Fig. 2D). On the contrary, BACHD/mGluR5^−/−^ exhibited a reduction in body weight as compared to BACHD mice (One-way ANOVA, 2 months, F(_3,31_) = 9.531, p < 0.0001, and 6 months F(_3,31_) = 26.14, p < 0.0001; Kruskal–Wallis, 12 months, p < 0.0001). Notably, the weight measurements of the double mutant mice were statistically equal to WT mice (One-way ANOVA 2 and 6 months, p > 0.9999; Kruskal–Wallis, 12 months p = 0.9279, Fig. 2B–D). Taken together, these data suggest that mGluR5 deletion restore body weight to control levels.

Next, we decided to verify the adiposity indices in all tested groups. For that, we performed the measurement of the visceral fat in the EAT, MAT and RPAT fat pads, at 6 and 12 months of age, which correspond to the initial and late phases of HD in BACHD mice^27^. Notably, WT mice and BACHD mice did not present statistical differences in the wet weight of adipose tissues, except in the RPAT at 6 months of age (One-way ANOVA, RPAT F(_3,11_) = 9.990, p = 0.0297) (Fig. 3E). However, the adiposity index is not altered, leading us to suggest that this parameter is not affected by mHTT (Fig. 3G, H). Importantly, our results demonstrated that at 6 months, the wet weight of all fat pads and the adiposity were reduced as compared BACHD/mGluR5^−/−^ and BACHD mice (One-way ANOVA, EAT F (_3,11_) = 17.82, p = 0.0004, RPAT F(_3,11_) = 9.990, p = 0.0041, and adiposity index, F (_3,11_) = 14.3, p = 0.0004; Kruskal–Wallis, MAT, p = 0.0266) (Fig. 3A,C,E,G). At 12 months, these differences were just observed in MAT, followed by a tendency of RPAT (One-way ANOVA, MAT F(_3,21_) = 5.515, p = 0.0231; Kruskal–Wallis, RPAT, p = 0.0524) (Fig. 3D,F). Nevertheless, visceral adiposity of BACHD/mGluR5^−/−^ is only significantly different as compared to WT mice (Kruskal–Wallis, 12 months, p < 0.0051) (Fig. 3H). Thus, it is quite tempt to infer that the deletion of mGluR5 is decreasing the adiposity index in BACHD mice at 6 months of age.

### Absence of mGluR5 promotes changes in the adipokines levels in the adipose tissue of BACHD mice

**Figure 4:**
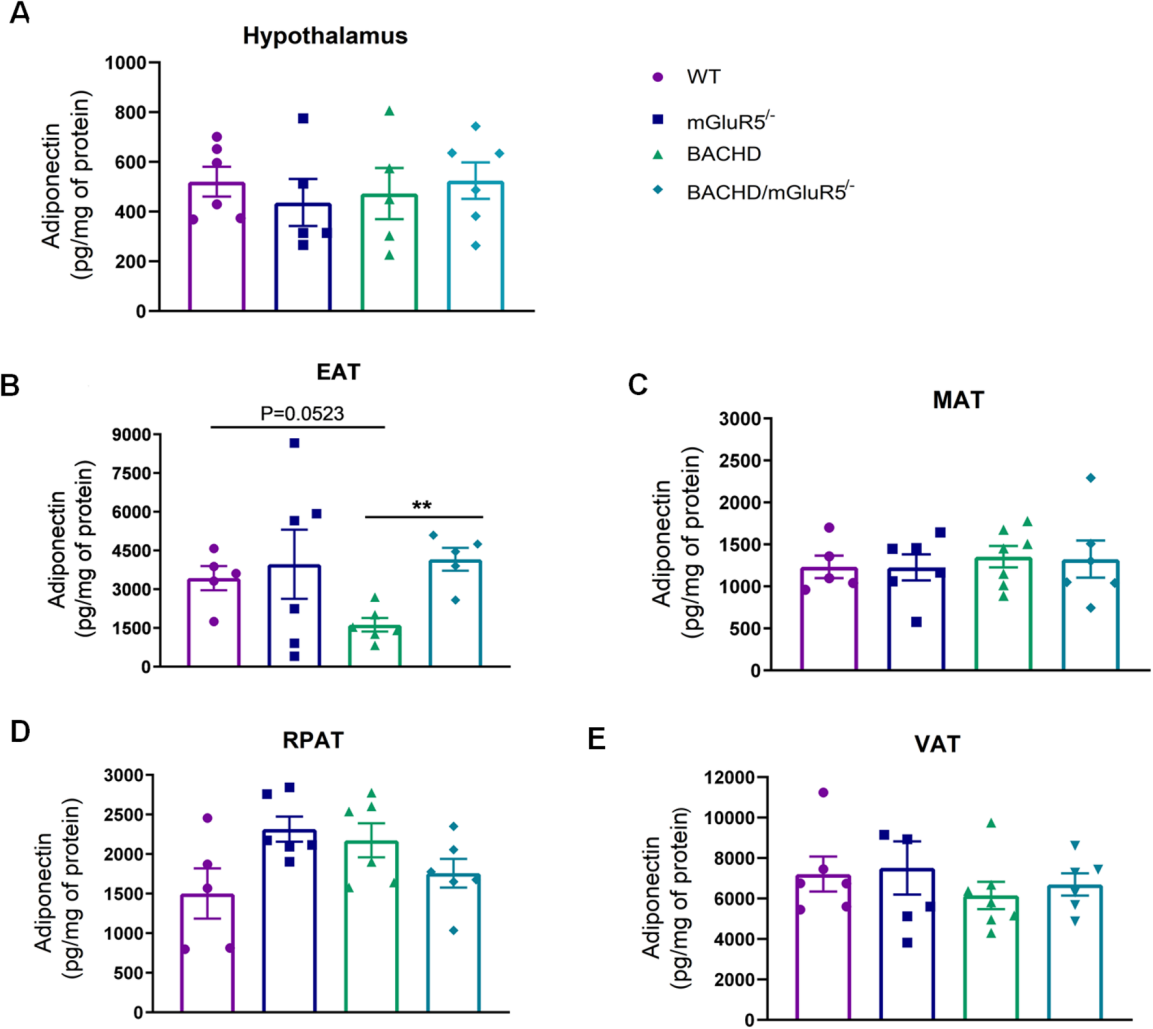
The deletion of mGluR5 restores adiponectin levels in the epididymal adipose tissue of BACHD mice. (**A**–**E**) Adiponectin levels in the hypothalamus, epididymal (EAT), mesenteric (MAT), retroperitoneal (RPAT) adipose tissues, and in the sum of visceral adipose tissues (VAT) of WT, mGluR5^−/−^, BACHD and BACHD/mGluR5^−/−^ mice at 12 months of age. Error bars represent the mean ± SEM; n = 5–7. One-Way ANOVA followed by Bonferroni post test (**A**, **C**–**E**), and One-Way Welch ANOVA followed by Games-Howell’s post test (**B**). * p < 0.05; ** p < 0.01.

Due to the importance of adipokines, such as leptin and adiponectin, in the context of energy and inflammatory balance, we investigated the concentration of these adipokines in the hypothalamus of 12-month-old mice (Fig. 4A) and also in the EAT, MAT, RPAT and in the sum of VAT in all tested groups from 6 and 12 months of age (Fig. 4B–E). Our results showed that at 6 months of age, there was no significant difference in the concentration of adiponectin between all genotypes in any of the analyzed tissues (Supplementary data, figure S1). However at 12 months of age, we observed in the EAT from BACHD mice, a tendency of decrease of adiponectin levels as compared to WT mice (Fig. 4B). Moreover, the deletion of mGluR5 in BACHD mice was able to normalize the adiponectin to control levels (One-Way Welch ANOVA, EAT W 8.785 (3.000, 9.133), p = 0.0047).

**Figure 5:**
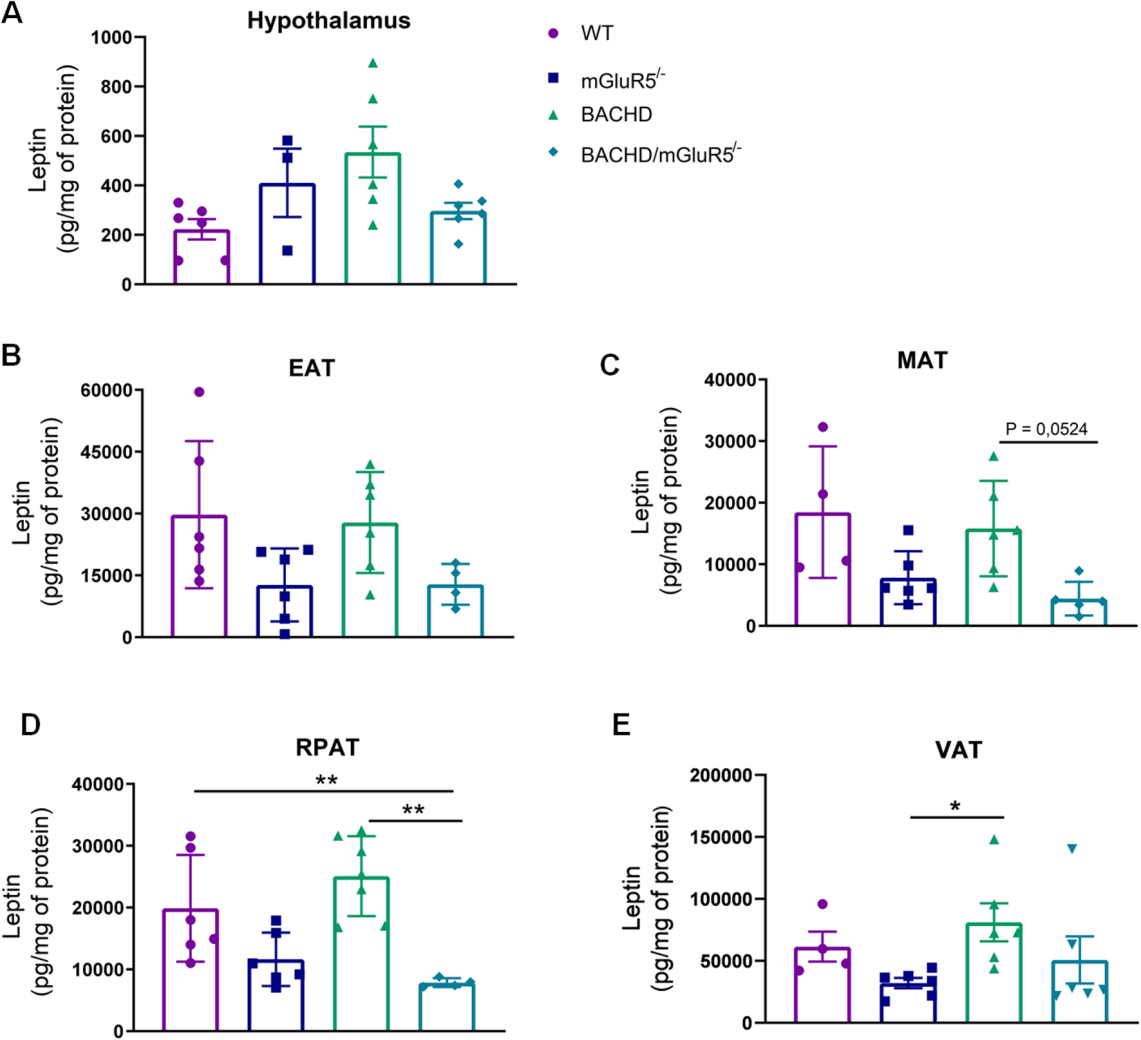
The absence of mGluR5 decreases leptin levels in BACHD mice at 12 months of age (**A**–**E**) Leptin levels in the hypothalamus, epididymal (EAT), mesenteric (MAT), retroperitoneal (RPAT) adipose tissue, and in the sum of visceral adipose tissues (VAT) of WT, mGluR5^−/−^, BACHD and BACHD/mGluR5^−/−^ mice at 12 months of age. Error bars represent the mean ± SEM; n = 4–7. One-Way Welch ANOVA followed by Games-Howell’s post test (**A**–**D**), and Kruskal–Wallis followed by Dunn’s post test (**E**). * p < 0.05; ** p < 0.01.

Next, leptin levels were increased in the VAT of BACHD mice as compared to WT mice at 6 months of age (One-Way ANOVA, MAT F(_3,10_) = 11, P = 0.0013) (Supplementary data, figure S2). However, at 12 months of age the leptin levels on BACHD mice were not statistical different from WT group in any of the adipose tissue tested (Fig. 5B–E). By contrast, we found that at 12 months of age leptin levels had a tendency of decreasing in MAT and RPAT of BACHD/mGluR5^−/−^ mice as compared to BACHD group (One-Way Welch ANOVA, MAT W 4.843 (3.000, 8.038), p = 0.0524, RPAT W 18.26 (3.000, 9.213), p = 0.0003) (Fig. 5C,D). Interestingly, in both analysis of hypothalamic adiponectin and leptin levels, we did not observe differences between double mutant and BACHD mice (Figs. 4A, 5A), which may indicate a peripheral modulation caused by the absence of mGluR5 receptor on these adipokines.

**Figure 6:**
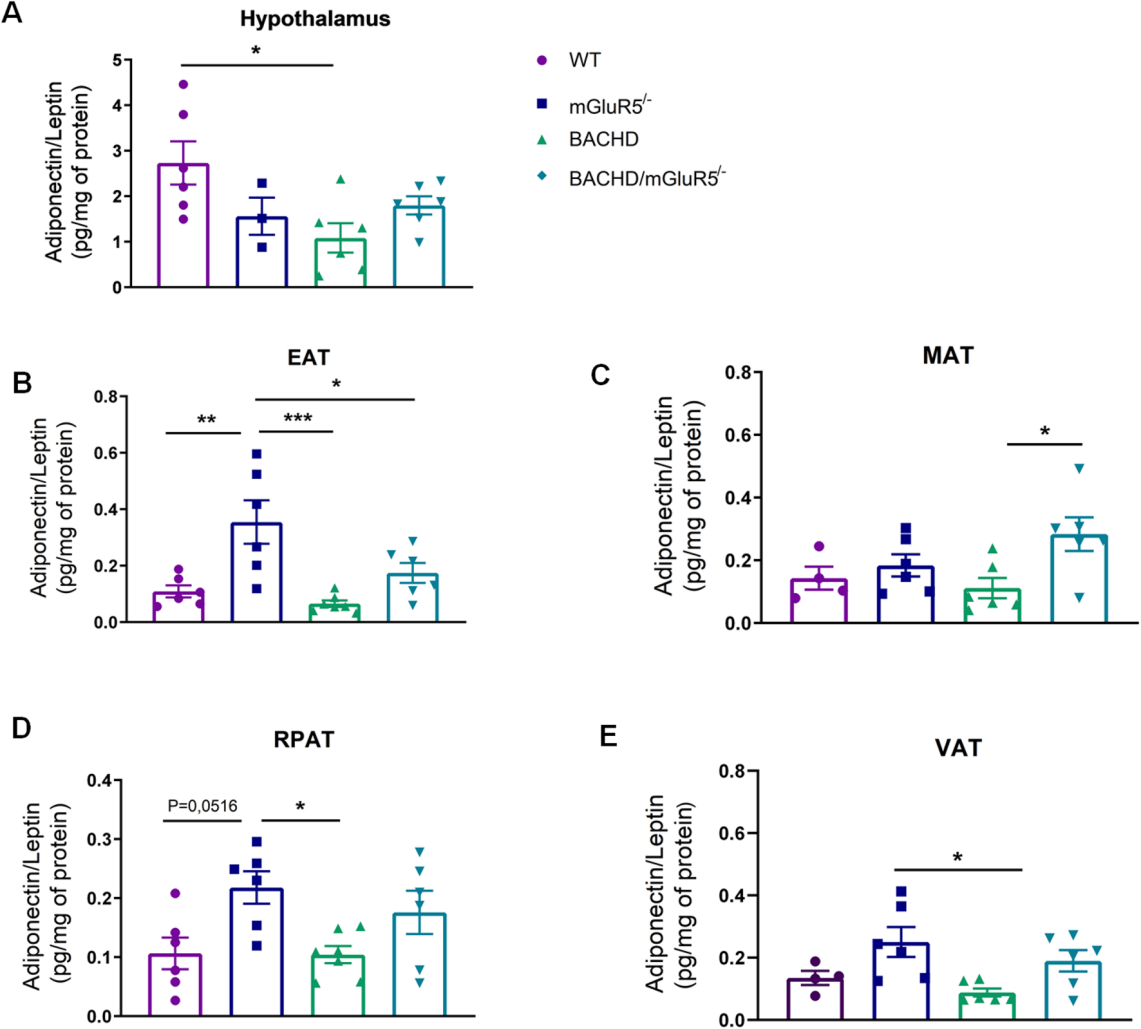
mGluR5 KO mice present increased adiponectin/leptin levels in several adipose tissue. (**A**–**E**) Adiponectin/leptin ratio in the hypothalamus, epididymal (EAT), mesenteric (MAT), retroperitoneal (RPAT) adipose tissue, and in the sum of visceral adipose tissues (VAT) of WT, mGluR5^−/−^, BACHD and BACHD/mGluR5^−/−^ mice, at 12 months of age. Error bars represent the mean ± SEM; n = 4–7. One-Way ANOVA followed by Bonferroni post-test (**A**–**D**) and Kruskal Wallis followed by Dunn’s post test (**E**). * p < 0.05; ** p < 0.01; ***p < 0.005.

Due to the antagonistic role of adiponectin and leptin in the obesity context, we decided to analyze the ratio of adiponectin/leptin at 6 and 12 months of age in all tested groups (Supplementary data, Figure S3 and Figure S6). In this milieu, at 6 months of age, there was no significant difference in the adiponectin/leptin ratio between WT and BACHD mice in all analyzed adipose tissues (Supplementary data, Figure S3). Our data showed that BACHD mice presented a decrease in the hypothalamic adiponectin/leptin ratio as compared to WT mice at 12 months of age (Fig. 6A). Moreover, adiponectin/leptin ratio in the mGluR5 KO mice is increased as compared to WT and BACHD mice in the EAT and RPAT (Fig. 6B, D) and increased as compared to BACHD in the VAT (Fig. 6E). Plus, at 12 months analyzing MAT, we found that the BACHD/mGluR5^−/−^ mice exhibited higher adiponectin/leptin ratio as compared to BACHD mice (One-way ANOVA, MAT F(_3,18_) = 3.425, p = 0.0394) (Fig. 6C). Notably, these data pointed out a possible mechanism involving mGluR5 in the regulation of these adipokines.

### The absence of mGluR5 promotes increased an anti-inflammatory profile in the double mutant mice

**Figure 7:**
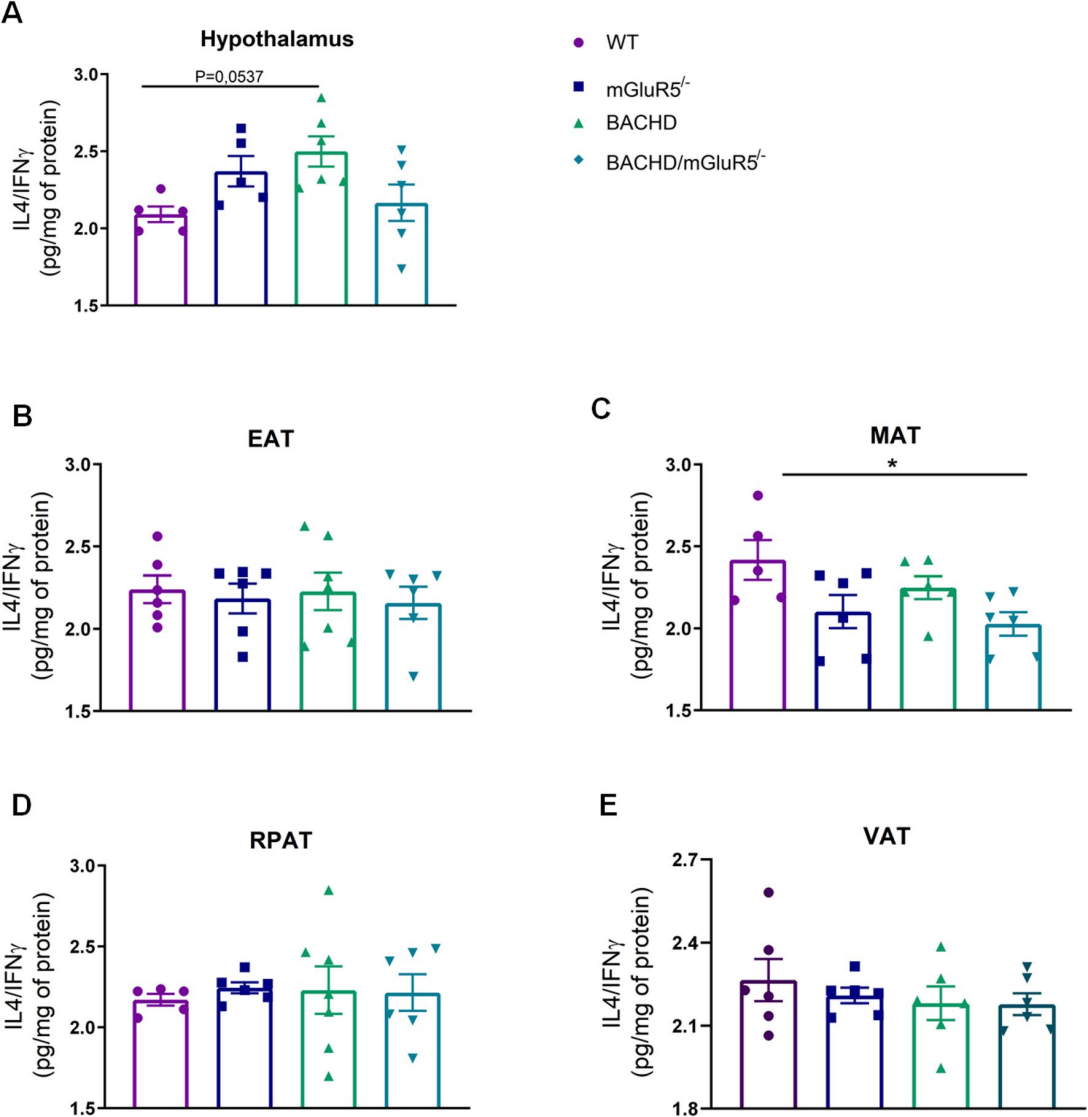
BACHD/mGluR5^−/−^ mice exhibit decreased IL-4/IFN ratio in the mesenteric adipose tissue. (**A**–**E**) IL-4/IFN ratio in the hypothalamus, epididymal (EAT), mesenteric (MAT), retroperitoneal (RPAT) adipose tissue, and in the sum of visceral adipose tissue (VAT) of WT, mGluR5^−/−^, BACHD and BACHD/mGluR5^−/−^ mice, at 12 months of age. Error bars represent the mean ± SEM; n = 5–7. One-Way ANOVA followed by Bonferroni's post-test (**A**, **C**), Kruskal Wallis followed by Dunn’s post test (**B**, **E**), and One-Way Welch ANOVA followed by Games-Howell’s post test (**C**). *p < 0.05.

**Figure 8:**
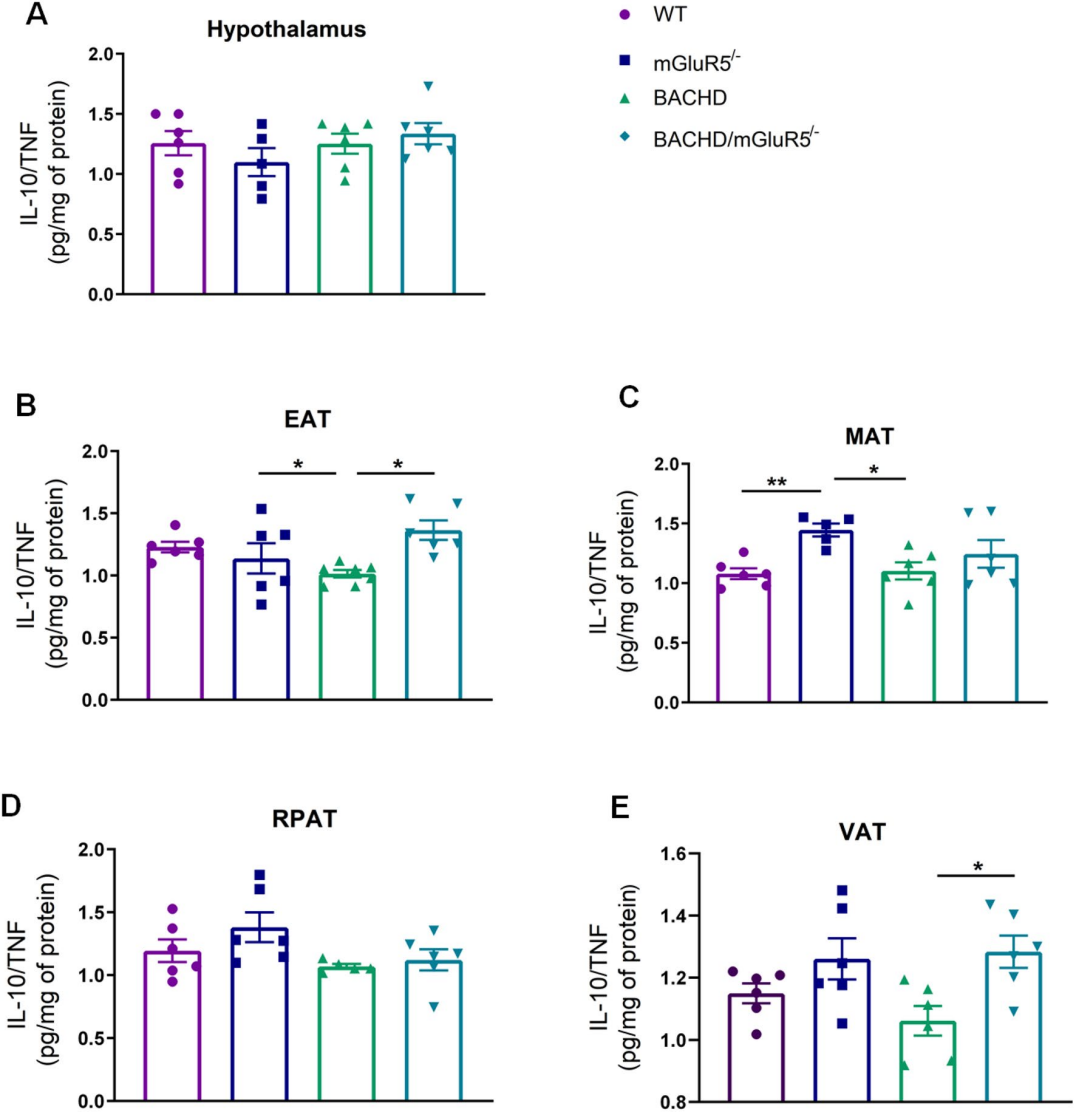
BACHD/mGluR5^−/−^ mice present an increase of IL-10/TNF ratio in the EAT, and in the sum of visceral adipose tissue. (**A**–**E**) IL-10/TNF ratio in the hypothalamus, epididymal (EAT), mesenteric (MAT), retroperitoneal (RPAT) adipose tissue, and in the sum of adipose tissues (VAT) of WT, mGluR5^−/−^, BACHD and BACHD/mGluR5^−/−^ mice at 12 months of age. Error bars represent the mean ± SEM; n = 5–7. One-Way ANOVA followed by Bonferroni post-test (**A**, **E**), and One-Way Welch ANOVA followed by Games-Howell’s post test (**B**, **C**, **D**). *p < 0.05.

In order to investigate the inflammatory state of all tested groups, we decided to measure the levels of pro and anti-inflammatory cytokines. At 6 and 12 months of age comparing WT and BACHD mice, our findings did not show remarkable differences between pro and anti-inflammatory cytokines in central and periphery tissues analyzed (Supplementary data, figures S4–7 and figures S10–13). However, our findings showed that BACHD/mGluR5^−/−^ mice presented an increase in both pro-inflammatory and anti-inflammatory cytokines as compared to BACHD and WT mice in all VAT and also in the hypothalamus at 6 and 12 months of age (Supplementary data, figures S4–7 and figures S10–13). Thus, in order to verify which cytokine, as well as the predominant immune response in the analyzed tissues, we examined ratios between IL-4/IFN and also IL-10/TNF. We found that there was no difference in all ratios between tested genotypes at 6 months of age (Supplementary Figures S8–9). However, at 12 months of age, BACHD/mGluR5^−/−^ mice showed a reduction in IL-4/IFN ratio as compared to WT mice in MAT (One-way ANOVA, F(_3,19_) = 3,416, p = 0.0384) (Fig. 7C), indicating a pro-inflammatory state in the MAT of these mice. However, this fact was not observed in the sum of the VAT or in all other tissues analyzed (Fig. 7A, B, D, E). Furthermore, in the hypothalamus of BACHD mice a higher IL-4/IFN ratio was observed as compared to WT mice (One-way ANOVA, F(_3,18_) = 3.687, p = 0.0314) (Fig. 7A). Regarding the ratio IL-10/TNF (Fig. 8A–E), we observed that BACHD/mGluR5^−/−^ mice showed a higher IL-10/TNF ratio as compared to BACHD mice in the EAT, and in the sum of VAT (One-Way Welch ANOVA, EAT W 8.373 (3.000, 10.79), p = 0.0040; One-way ANOVA, VAT F(_3,20_) = 4.096, p = 0.0203) (Fig. 8B,E). Therefore, these set of data may indicated that absence of mGluR5 may promote a balance in the inflammatory environment in the adipose tissue of BACHD mice.

## Discussion

**Figure 9:**
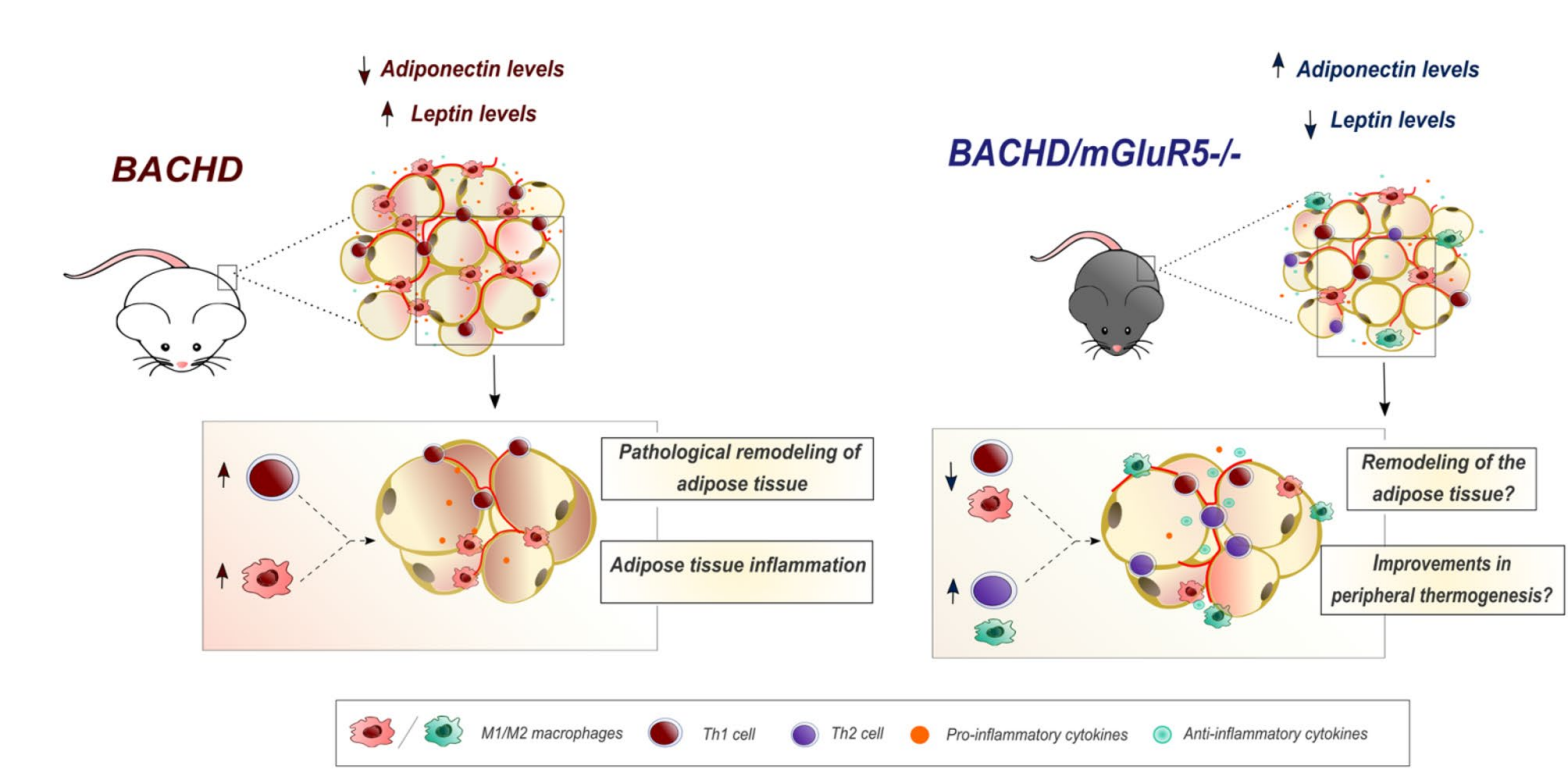
Proposed mechanism of mGluR5 absence in the improvements of obesity phenotype associated to BACHD mice. BACHD mice present an obesity phenotype associated with an inflammatory disturbance in the visceral white adipose tissue, leading to alterations in the peripheral adipokines. We hypothesized that the absence of mGluR5 signaling in the BACHD/mGluR5^−/−^ promoting an anti-inflammatory outcome in the visceral white adipose tissue, which may be associated with the modulation of macrophage polarization and T cells, and remodeling of the visceral white adipose tissue. Furthermore, reduction of inflammatory environment can regulate adipokine profile, and improving peripheral anorexigen signaling, contributing to regulate body weight in the double mutant mice.

The main idea of this work was to understand the part of mGluR5 in obesity by investigating how receptor ablation may regulate the altered metabolic profile of BACHD mice. Several authors have indicated that transgenic mice models of HD presenting full-length HTT display some obese features^27,30,32,34^. Elegant data postulated that HTT acting on hypothalamic circuits may induce increased appetite, insulin resistance, and body fat accumulation on BACHD mice^26,27^. It has been also shown that BACHD mice obese phenotype is associated with a hyperphagic behavior due to alterations in the monoaminergic circuitry, such as decreased hypothalamic expression of vesicular monoamine transporter 2 (VMAT2), which may be associated to an increase of the binge-like eating behavior^27,37,38^. Our data confirmed previous results showing that BACHD mice have increased body weight as compared to WT mice in all tested ages^27^. In addition, we showed that the genetic deletion of mGluR5 in BACHD mice leads to a reduction in body weight. It has been reported that mGluR5 signaling is a mediator of appetite and energy balance^14,15^. Of note, a previous data showed that the blockade of mGluR5 may decrease anxiety behavior through serotonergic transmission, likely an increase in 5-HT release and the subsequent stimulation of 5-HT_2A/2C_ receptor^39^. Moreover, interesting study showed that 5-HT_2C_ receptor activation inhibits appetitive and consummatory components of feeding in mice^40^. Thus, although speculative, we propose that the decrease on body weight gain observed in the double mutant mice may be associated with the disinhibition of the serotonin release, which may act on the hypothalamus, reducing food consumption and/or increasing energy expenditure^41^ (Fig. 9). Nevertheless, herein, we did not evaluate the food intake, which is a limitation of our work. The decrease of food intake by the absence of mGluR5 may not be enough to explain the reduction in the body mass observed in the double mutant mice, especially at 6 and 12 months of age, as alterations in BACHD mice feeding behavior are only reported in a limited period of time, just before the establishment of metabolic disturbances^27^.

It has been reported that BACHD mice present elevated levels of hypothalamic and serum leptin, which may indicate resistance to this hormone^27^. It is well known that the amount of fat mass is directly related to leptin secretion, and that the increased body mass is also associated with a decrease in adiponectin levels^42,43^. Furthermore, adiponectin/leptin ratio has been suggested as a marker of adipose tissue dysfunction^44^. We observed that BACHD mice have a reduced hypothalamic adiponectin/leptin ratio and mGluR5 KO mice present increased visceral fat adiponectin/leptin ratio. Moreover, the deletion of mGluR5 increased the adiponectin/leptin ratio in the MAT of adipose tissue of BACHD mice. In addition, the deletion of mGluR5 in BACHD mice decreased visceral adiposity and wet weight of all fat pads evaluated, at 6 months, as well as the wet weight of MAT at 12 months. Although our experiments cannot fully elucidate this point, our data suggest a possible association between the absence of mGluR5 and the regulation of those adipokines through adipose tissue modulation in the BACHD mice. Leading in consideration that increased body mass may decrease adiponectin levels^42,43^, and that at physiological conditions, adiponectin promotes an increase in peripheral thermogenesis and energy expenditure in a synergic way with leptin^43,45^, we prior hypothesized that the absence of mGluR5 modulate adiposity in the long term, by increasing adiponectin levels in adipose tissue and improving thermogenesis, promoting weight loss due to an increase in the energy expenditure. In parallel, these improvements may decrease the peripheral leptin levels, reducing the effects of leptin resistance, promoting a peripheral synergic thermogenic effect with adiponectin (Fig. 9). Although speculative, this mechanism may produce substantial outcome on controlling the body weight in the long term. Importantly, our data did not allow us to infer about a prospective central effect.

Visceral pro-inflammatory environment is closely associated with systemic low grade chronic inflammation, as well as with adipokines dysfunction and endocrine disturbance in obesity^46,47^. In this context, recent work showed that BACHD mice also exhibited immune changes in peripheral organs^31^. Also previous study showed increased IL-4 plasma levels of HD patients at the moderate stage of the disease, suggesting that the increase of this cytokine may reflect an adaptive response to chronic immune activation^33^. Thus, we also aimed to investigate the profile of cytokines in the adipose tissue and the hypothalamus of tested genotypes by calculating the ratios between IL-4/INF and IL-10/TNF. Importantly, IFN inhibits the production of IL-4 in macrophages, reducing the differentiation of CD4+ naive T cells into type 2 helper T cells, which have an anti-inflammatory role in the context of obesity^45^. Likewise, IL-10 has an anti-inflammatory action, suppressing TNF production^48–51^. In our data, BACHD mice presented a tendency of increasing IL-4/INF levels (p = 0.0537) as compared to WT mice, indicating a pro-inflammatory central milieu. According to our data, mGluR5 did not present changes in IL-4/INF levels in mostly of the tested tissues. However, literatures shows that the negative modulation of mGluR5 by some drugs is able to decrease the transcription of NF-κB in T cell, TNFα, IL-12p70 and INFγ levels in EAT, which may reduce pro-inflammatory responses mediated by these cells^15,52^. Moreover, our data indicated that the absence of mGluR5 is associated to a higher IL-10/TNF ratio in the adipose tissue, which may indicate an anti-inflammatory tendency. Importantly, adipocytes produce several cytokines, but do not express mGluR5^53,54^. However, some studies demonstrated that activation of mGluR5 is important for the polarization and differentiation of macrophages and T lymphocytes^17,18^. Although we did not show the predominant cell population responsible for producing the cytokines modulated by the absence of mGluR5 in the adipose tissue of the double mutant mice, we suggest that the downregulation of mGluR5 possibly modulate the activation of macrophages and T cells in obese adipose tissue, which might be associated with the remodeling of the adipose tissue and influences the production of adipokines (Fig. 9). However, to validate these assumptions a deeper investigation will be required.

It is important to point out the limitations of this study. First, our results correspond to preliminary findings due the sample size for most of our analysis. The small n value for transgenic mice may be explained, at least in part, by the challenge involved in the bread and maintenance of transgenic mice. Importantly, the employed model doesn’t allow us to infer about translational mechanisms. Furthermore, we did not measure parameters as feeding behavior, thermogenic parameters, sex specific differences, as well as the predominant cell population responsible for producing the cytokines modulated by the absence of mGluR5 in the adipose tissue. Finally, some evidence show that mGluR5 ablation may affect gut microbiota composition, aging, neurodegeneration, sex-dependent behavior and cognitive functions^55–57^. Thus, given the importance of those factors in the metabolism and obesity, as well as in the progress of a neurodegenerative environment, a deeper investigation of the central mechanisms of energy balance, and cognitive function in our model is needed in order to elucidate how peripheral metabolic and immunomodulatory events promoted by absence of mGluR5, may modulate these central highpoints.

## Conclusion

Our main contribution was to show the effects of the deletion of mGluR5 in BACHD mice focusing on metabolic homeostasis and inflammatory parameters. The absence of mGluR5 reduced weight gain and visceral adiposity in BACHD mice, promoting an increase in adiponectin/leptin ratio, which may be associated with an anti-inflammatory environment in the adipose tissue. Thus, our preliminary findings indicate that deletion of mGluR5 may be associated with the modulation of the adipose tissue, which reveals a strong improvement in the peripheral effects related to the obesity phenotype observed in BACHD mice. Nevertheless, the implications of these events at CNS remain poorly elucidated. Therefore, further studies using other genetic models of obesity and also female mice, as sex differences influence immune response, are needed in order to investigate the ability of the negative modulation mGluR5 on promoting reduction of obesity and inflammation. Therefore, the investigation of pharmacological targets aiming mGluR5 to control the energy balance and inflammatory pathways may be interesting in the context of obesity.

## Supplementary Information

Supplementary Information.

## Abbreviations

mGluR5: Metabotropic Glutamate Receptor 5
CNS: Central Nervous System
EAT: Epididymal adipose tissue
RPAT: Retroperitoneal adipose tissue
VAT: Visceral adipose tissues
DIO: Diet-Induced-Obesity
HFD: High-fat-diet
HD: Huntington’s disease
TNF: Tumor Necrosis Factor
IFN-γ: Interferon gamma
IL: Interleukin
BACHD mice: Bacterial artificial chromosome (BAC)-mediated transgenic mouse model
mGluR5^−^^/^^−^: Metabotropic Glutamate Receptor 5 knockout mice
BACHD/mGluR5^−^^/^^−^: Double mutant mice
NAM: Negative allosteric modulator
KO: Knockout
