# Supplementary material for: Metabotropic glutamate receptor 5 knockout rescues obesity phenotype in a mouse model of Huntington’s disease
Source: Sci Rep. 2022 Apr 4;12:5621. doi: 10.1038/s41598-022-08924-4 (PMC8980063; doi:10.1038/s41598-022-08924-4)
Supplement: Supplementary file 1 — Supplementary Information. [file 41598_2022_8924_MOESM1_ESM.doc]

**Metabotropic glutamate receptor 5 knockout rescues obesity phenotype in a mice model of Huntington´s disease**

Rebeca P. M. Santos^a^, Roberta Ribeiro^a^, Talita H. F. Vieira^c^, Rosaria D. Aires^a^, Jessica M. de Souza^c^, Bruna S. Silva^b^, Anna Luiza D. Lima^a^, Antônio Carlos P. de Oliveira^a^, Helton J. Reis^a^, Aline S. de Miranda^b^, Erica M. L. Vieira^a^, Fabiola M. Ribeiro^c*^ and Luciene B. Vieira^a^**

**Supplementary data**

**Genotyping**

Genotyping was performed by chain polymerase reaction (PCR). The samples were obtained by toe cliping at the 10^th^ day post-birth, and the samples were stored at -80ºC. For DNA extraction, the samples were homogenized in a solution containg water for injection; sodium dodecyl sulfate (10%); Tris-EDTA (50x (ethylenediaminetetraacetic acid (EDTA) pH 8.0 (0.5 mol.L-1); Tris (hydroxymethyl) aminomethane hydrochloride (Tris HCl) pH 8.0 (1.0 mol.L-1) and water for injection)); NaCl (2.5 mol.L-1) and Proteinase K. Lysates were pre incubed (55ºC, 14-16 hours), centrifuged (15871*g*, 10 min), and isopropanol was added to the supernatant, that was again centrifuged (15871*g*, 10 min). The pellet was obtained and, after dry, processed with Tris-EDTA (0,5X) at 37ºC (1 h), and stored at -20ºC. Samples were mixed with a buffer compound with water for injection; dNTPs (10.0 mM); DMSO (5%); the identification primer for BACHD or mGluR5^-/-^, mGluR5^+/-^, and mGluR5^+/+^, (10.0 nM) (**Table S1**) and buffer provided by the enzyme manufacturer (10x); betaine (5 mol.L-1) and 0.60 units of the enzyme Taq DNA polymerase (Invitrogen). After, samples were submitted to a PCR thermal cyclers (For BACHD samples: 94ºC, 5min, 37 cycles; 94ºC, 20 seconds, 57ºC, 20 seconds, 70ºC, 90 seconds, 72ºC, 5 min; For samples from mGluR5^-/-^, mGluR5^+/-^, and mGluR5^+/+^: 94ºC, 3min, 35 cycles; 94ºC, 45 seconds, 60ºC, 45 seconds, 72ºC, 2 min and 45 seconds). Finally, electrophoresis was performed (100v, 22 min) in a agarose gel at 2% (0.5X of Tris-Acetate-EDTA (TAE) buffer solution; agarose, and 10 μL of SYBR® Safe (Life Technologies), and images were obtained by imageQuant LAS 4000 (GE Healthcare Life Sciences).

**Table S1 – Primers used for PCR**

| **Primers** | **Sequency** | **Amplicon (pb)** | **% GC** |
| --- | --- | --- | --- |
| ***HTT-M4F*** | TGAATTGTACAGCCGATGGA | 157 | 53,4 |
| ***HTT-M4R*** | CCGTAGTTCTGTCAGCGTCA | 157 | 57,5 |
| ***mGluR5 (WT)*** | CACATGCCAGGTGACATCAT |  |  |
| ***mGluR5 (WT, reverse)*** | CCATGCTGGTTGCAGAGTAA | 442 | 50,0 |
| ***mGluR5 (mutant, reverse)*** | CACGAGACTAGTGAGACGTG | 650 | 55,0 |

**Note:** The primers used for identification of BACHD (*HTT-M4F*, and *HTT-M4R*), homozygous mutant (mGluR5^-/-^), heterozygous (mGluR5^+/-^) and homozygous wild-type (mGluR5^+/+^) animals were obtained from the Jackson Laboratory, and developed from the sequence present in GenBank under identifiers NM_010414.2, and NM_001081414.2, respectively.

**
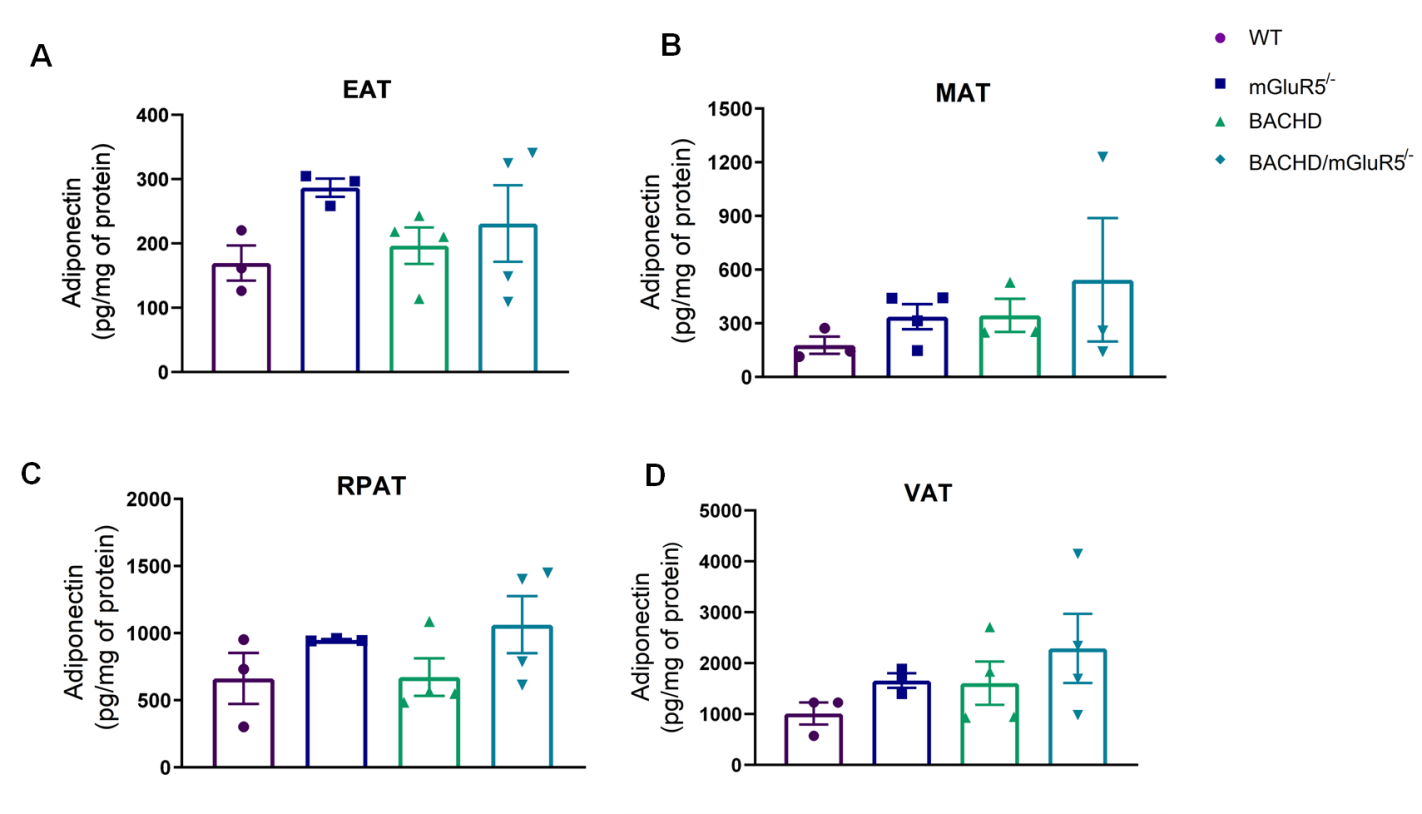
**

**Figure S1 – At 6 months of age, BACHD/mGluR5^-/-^ mice not presented alterations in adiponectin levels.** (**A**-**D**) Adiponectin concentration in epididymal (EAT), mesenteric (MAT), retroperitoneal (RPAT) adipose tissue, and in the sum of visceral adipose tissue (VATs) of WT, mGluR5, BACHD and BACHD/mGluR5^-/-^ mice, at 6 months of age. Error bars represent the mean ± SEM; n = 3-4. Krustal-Wallis followed by Dunn’s post hoc test.

**
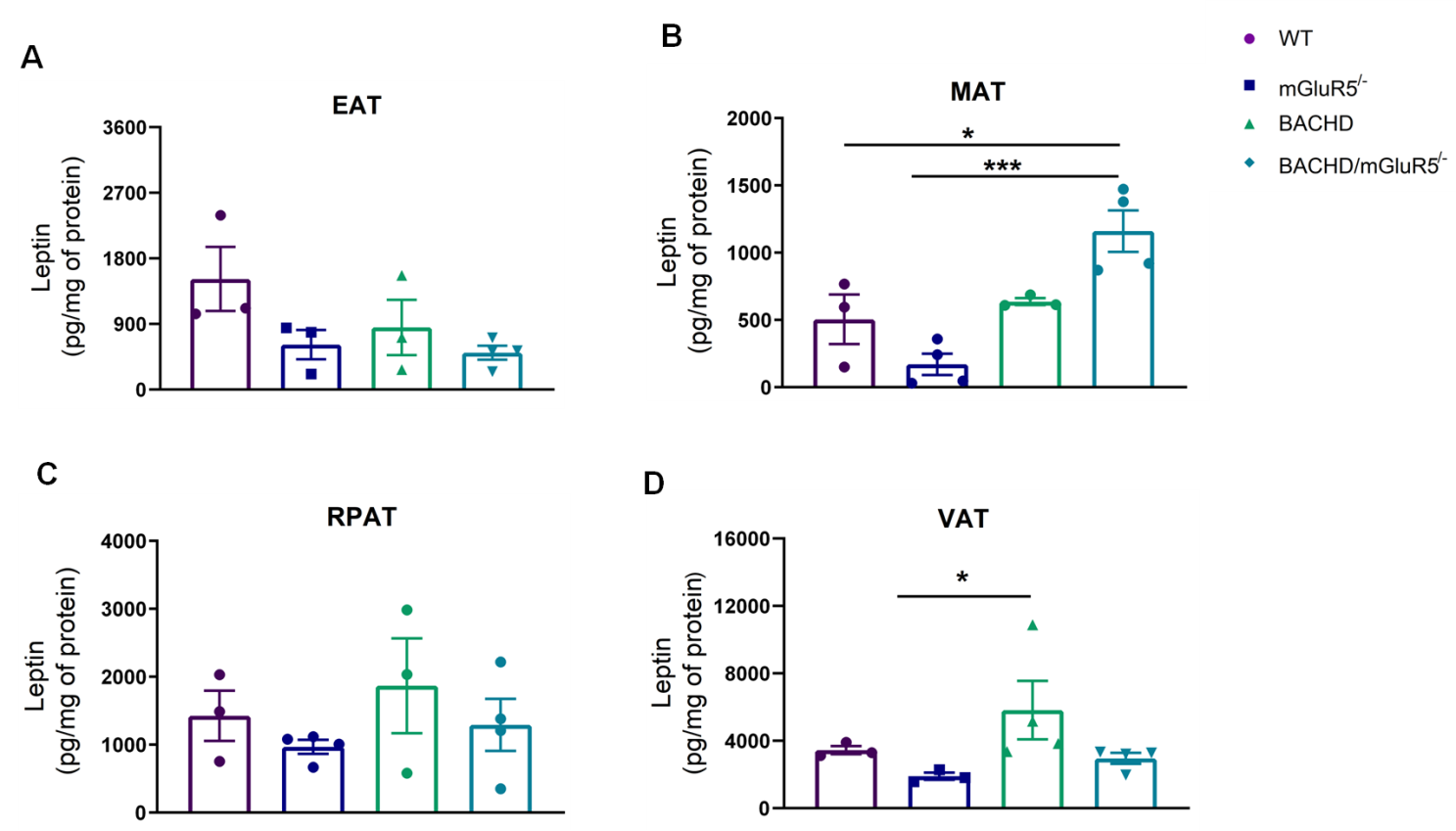
**

**Figure S2 – The mesenteric adipose tissue of BACHD/mGluR5^-/-^ mice presented an increase in leptin levels, at 6 months of age.** (**A**-**D**) Leptin concentration in the hypothalamus, epididymal (EAT), mesenteric (MAT), retroperitoneal (RPAT) adipose tissue, and in the sum of visceral adipose tissues (VAT) of WT, mGluR5, BACHD and BACHD/mGluR5^-/-^ mice, at 6 months of age. Error bars represent the mean ± SEM; n = 3-4. One-Way ANOVA followed by Bonferroni post hoc test (**A**-**C**) and Krustal-Wallis followed by Dunn’s post hoc test (**D**). *P <0.05; ***P <0.001.


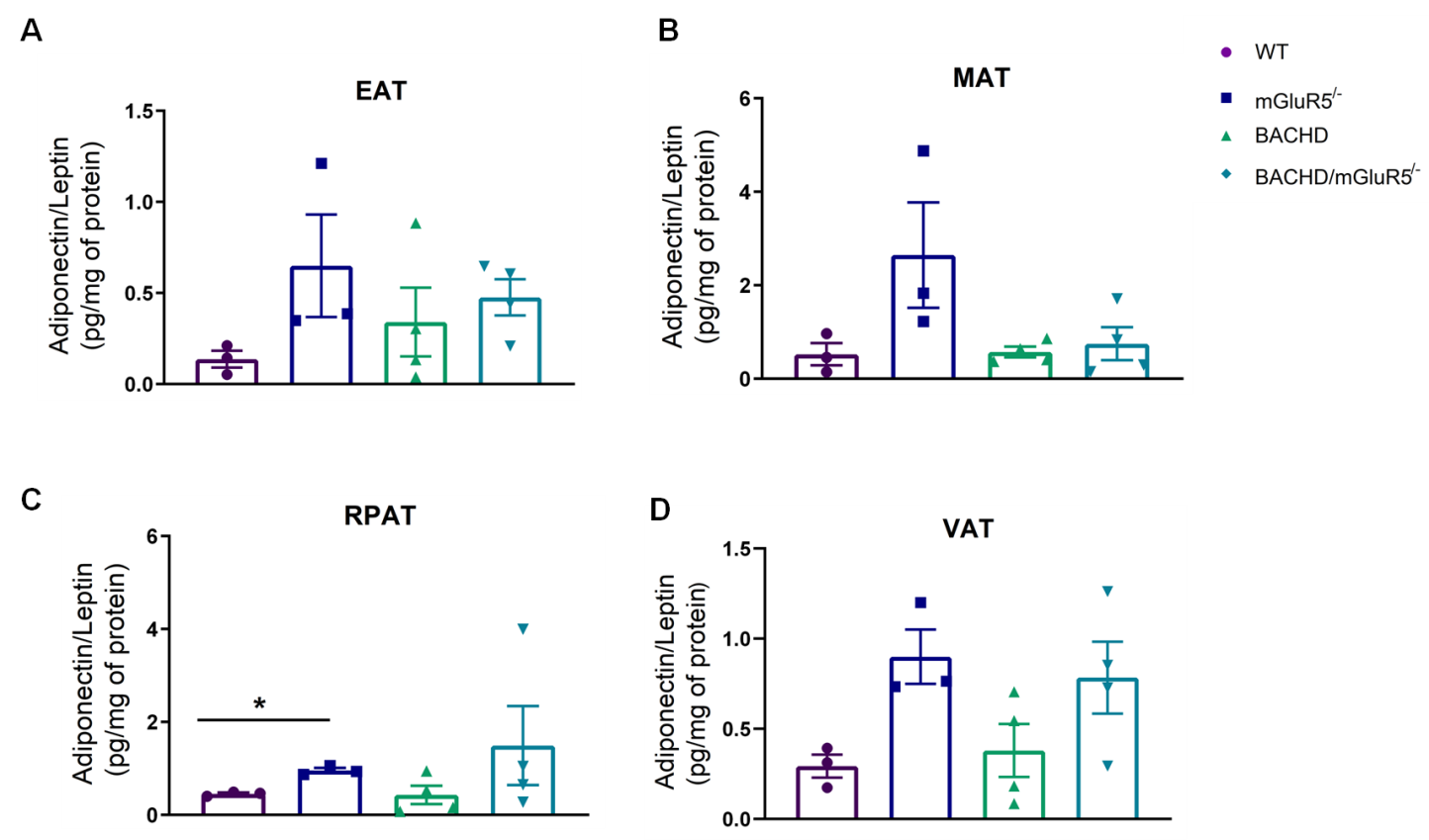


**Figure S3 – At, 6 months of age, the Adiponectin/leptin ratio is not increased in BACHD/mGluR5^-/-^** **mice.** (**A**-**E**) Adiponectin/leptin ratio in the hypothalamus, epididymal (EAT), mesenteric (MAT), retroperitoneal (RPAT) adipose tissue, and in the sum of visceral adipose tissues (VAT) of WT, mGluR5, BACHD and BACHD/mGluR5^-/-^ mice, at 6 months of age. Error bars represent the mean ± SEM; n = 3-4. One-Way ANOVA followed by Bonferroni post-test (**B, C,** and **E**) and One-Way Welch ANOVA followed by Games-Howell’s post hoc test (**D**). * P <0.05.


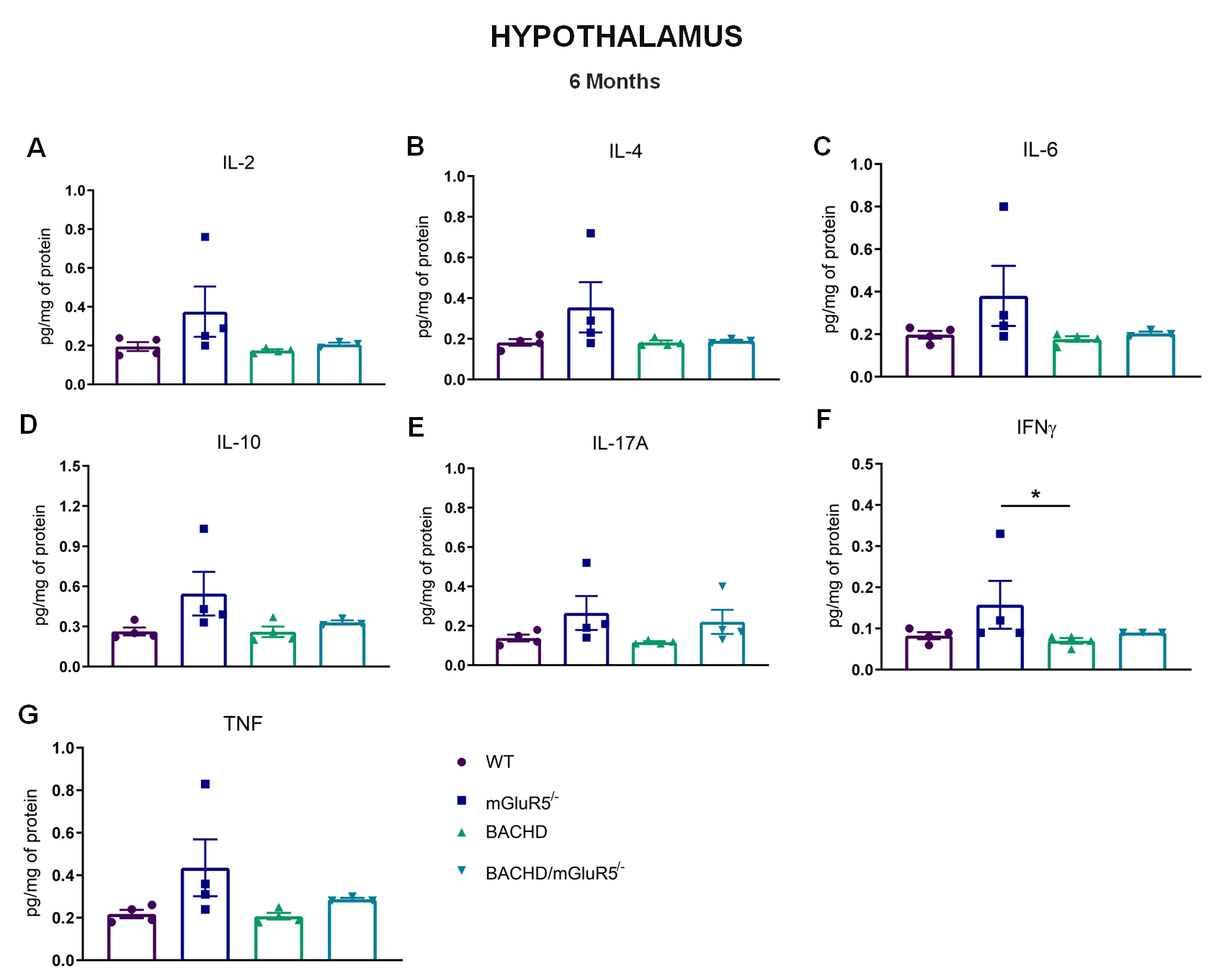


**Figure S4- The levels pro-inflammatory and anti-inflammatory cytokines in hypothalamus is not altered in the BACHD/mGluR5^-/-^ mice at 6 months of age.** (**A**-**G**) Hypothalamic measurement, by cytometric bead assay (CBA), of the individual levels of IL-2, IL-4, IL-6, IL-10, IL-17A, INFγ, and TNF of WT, mGluR5, BACHD and BACHD/mGluR5^-/-^ mice, at 6 months of age. Error bars represent the mean ± SEM; n = 3-4. Krustal-Wallis followed by Dunn’s post test (**A**, **C**, **D**, **F**, and **G**), and One-Way Welch ANOVA followed by Games-Howell’s post hoc test (**B** and **E**). *P <0.05.


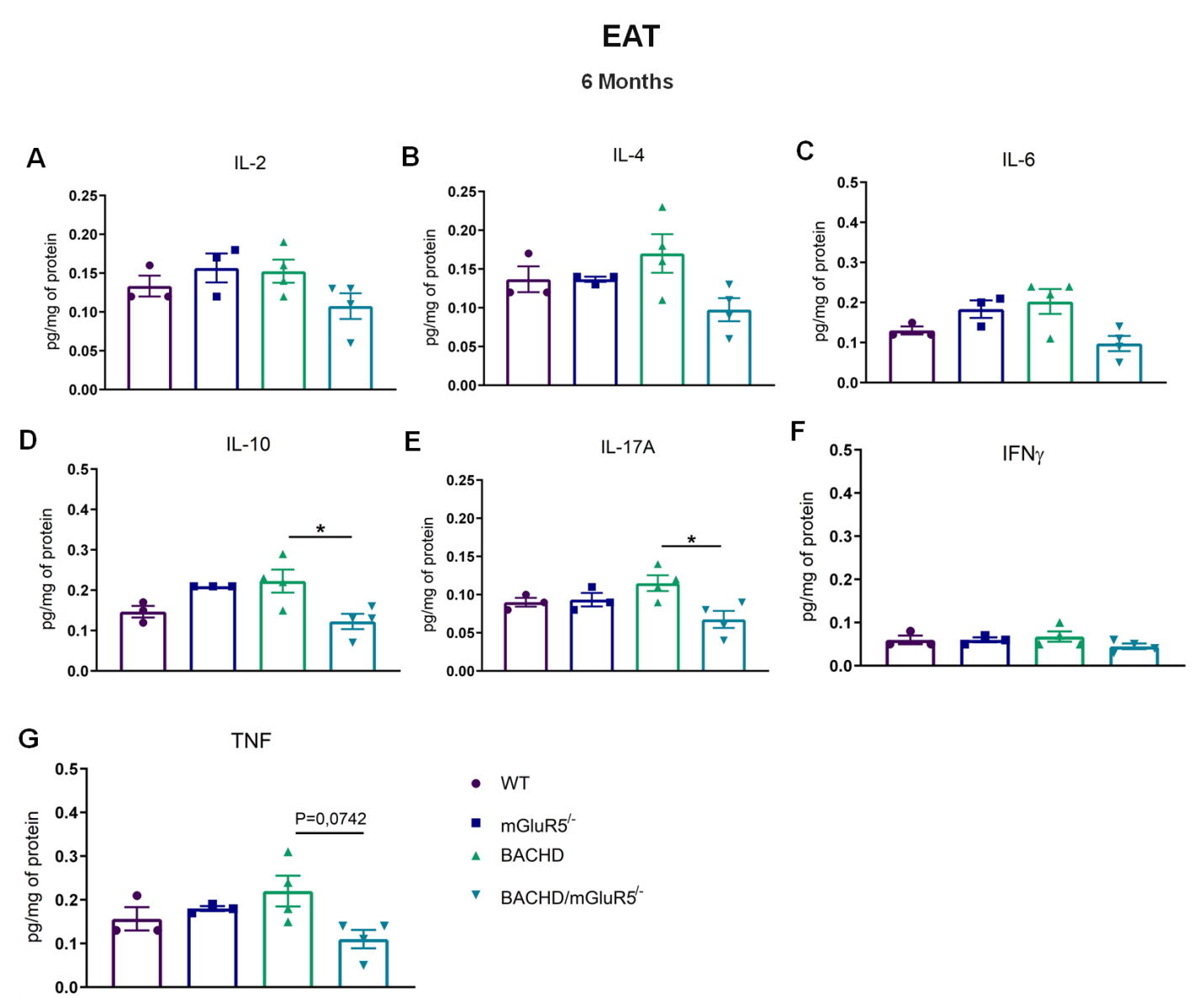


**Figure S5- BACHD/mGluR5^-/-^ mice presented a decrease in IL-10, and IL-17A levels in epididymal adipose tissue (EAT) at 6 months of age.** (**A**-**G**) Measurement, by cytometric bead assay (CBA), of the individual levels of IL-2, IL-4, IL-6, IL-10, IL-17A, INFγ, and TNF in EAT of WT, mGluR5, BACHD and BACHD/mGluR5^-/-^ mice, at 6 months of age. Error bars represent the mean ± SEM; n = 3-4. Krustal-Wallis followed by Dunn’s post hoc test (**A**, **B**, **C**, and **G**), and One-Way ANOVA followed by Bonferroni post hoc test (**D, E** and **F**). *P <0.05.


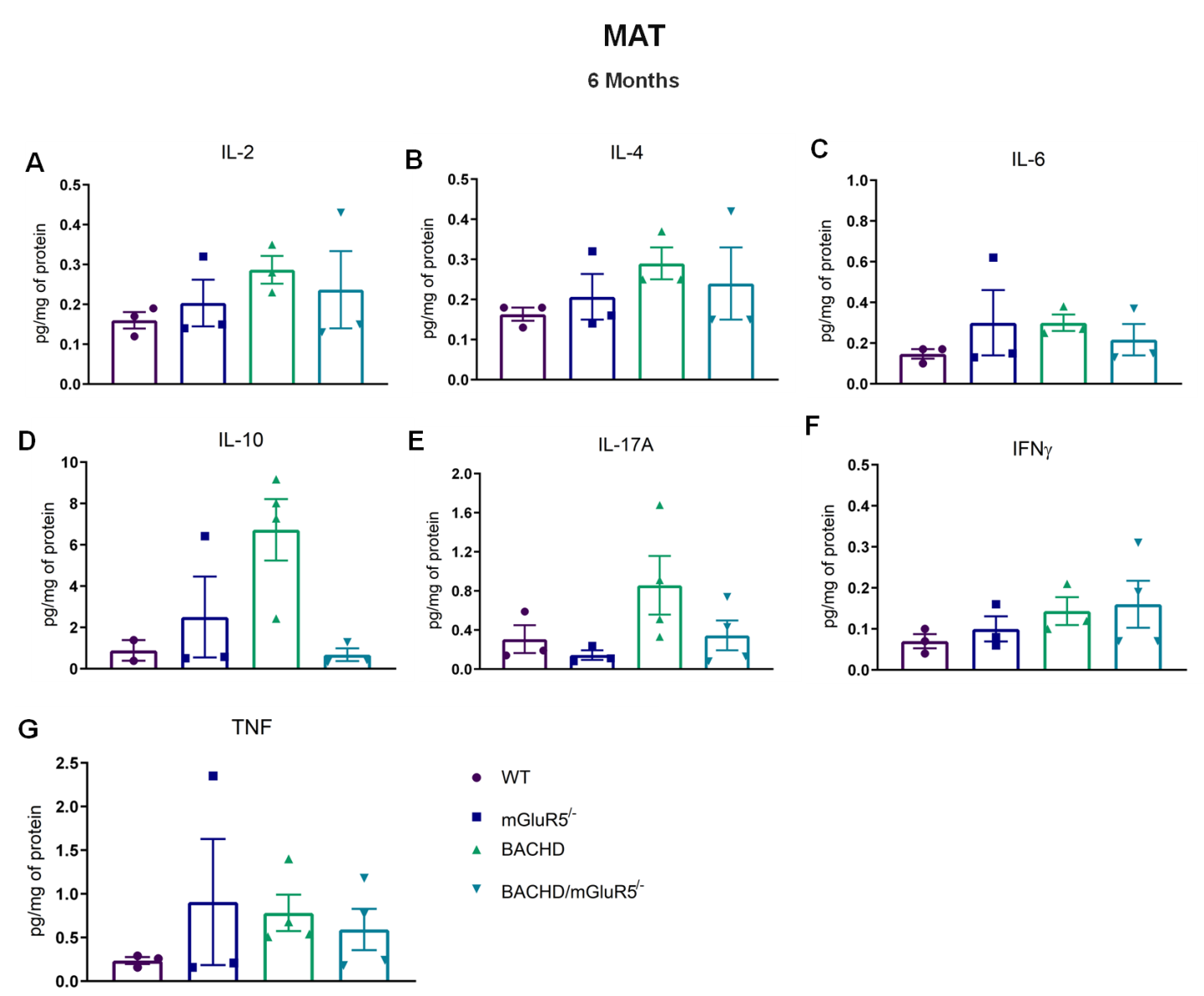


**Figure S6- At 6 months, the levels pro-inflammatory and anti-inflammatory cytokines in mesenteric adipose tissue (MAT) is not altered in the interesting groups.** (**A**-**G**) Measurement, by cytometric bead assay (CBA), of the individual levels of IL-2, IL-4, IL-6, IL-10, IL-17A, INFγ, and TNF in MAT of WT, mGluR5, BACHD and BACHD/mGluR5^-/-^ mice, at 6 months of age. Error bars represent the mean ± SEM; n = 3-4. One-Way Welch ANOVA followed by Games-Howell’s post hoc test (**A**), Krustal-Wallis followed by Dunn’s post test (**B**, **C**, and **G**), and One-Way ANOVA followed by Bonferroni post hoc test (**D, E** and **F**).


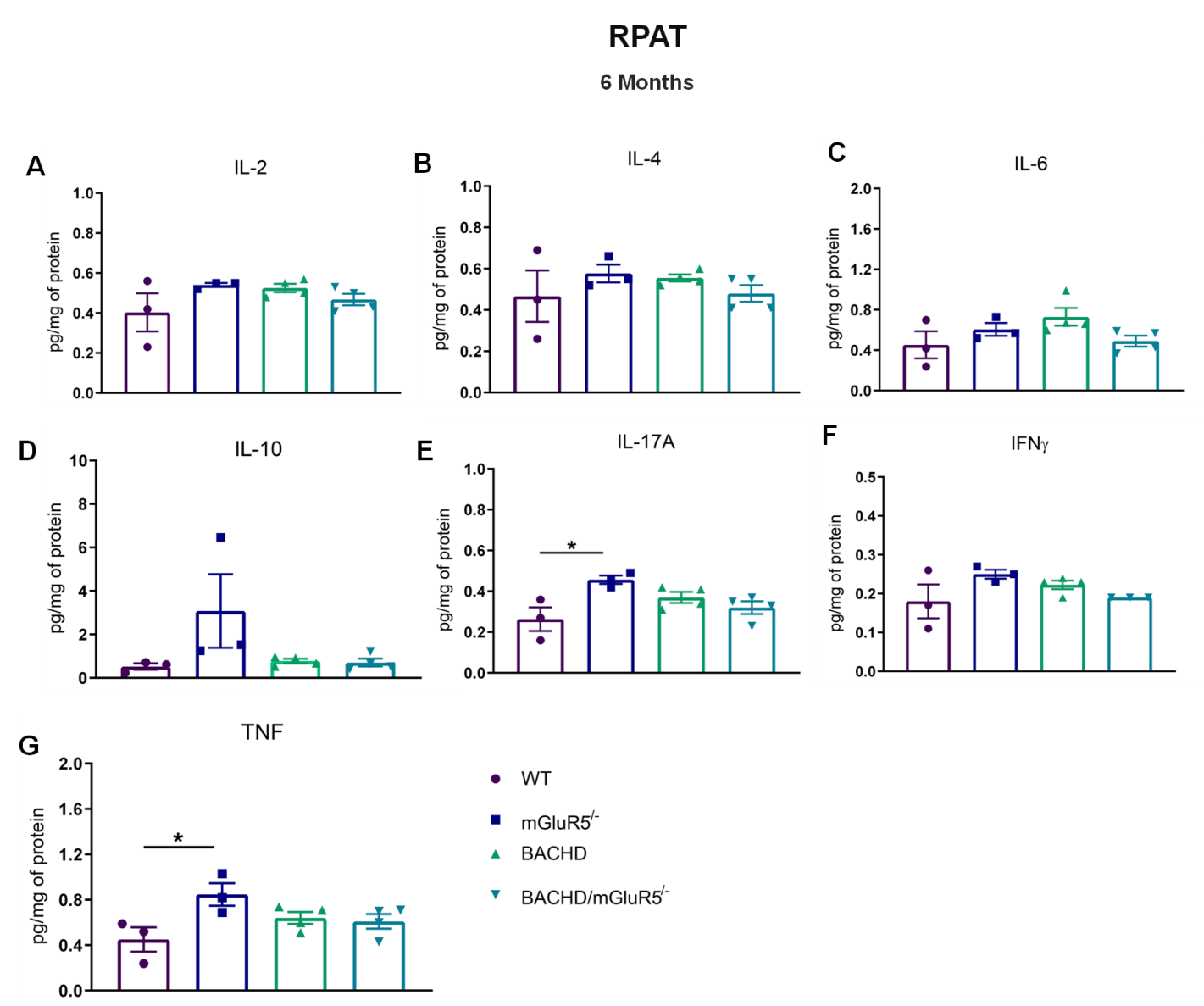


**Figure S7- The levels pro-inflammatory and anti-inflammatory cytokines in retroperitoneal adipose tissue (RPAT) is not altered in the BACHD/mGluR5^-/-^ mice at 6 months of age.** (**A**-**G**) measurement, by cytometric bead assay (CBA), of the individual levels of IL-2, IL-4, IL-6, IL-10, IL-17A, INFγ in RPAT of WT, mGluR5, BACHD and BACHD/mGluR5^-/-^ mice, at 6 months of age. Error bars represent the mean ± SEM; n = 3-4. Krustal-Wallis followed by Dunn’s post hoc test (**A**, **B**, and **F**), One-Way Welch ANOVA followed by Games-Howell’s post hoc test (**D**), and One-Way ANOVA followed by Bonferroni post hoc test (**C, E** and **G**). *P <0.05.


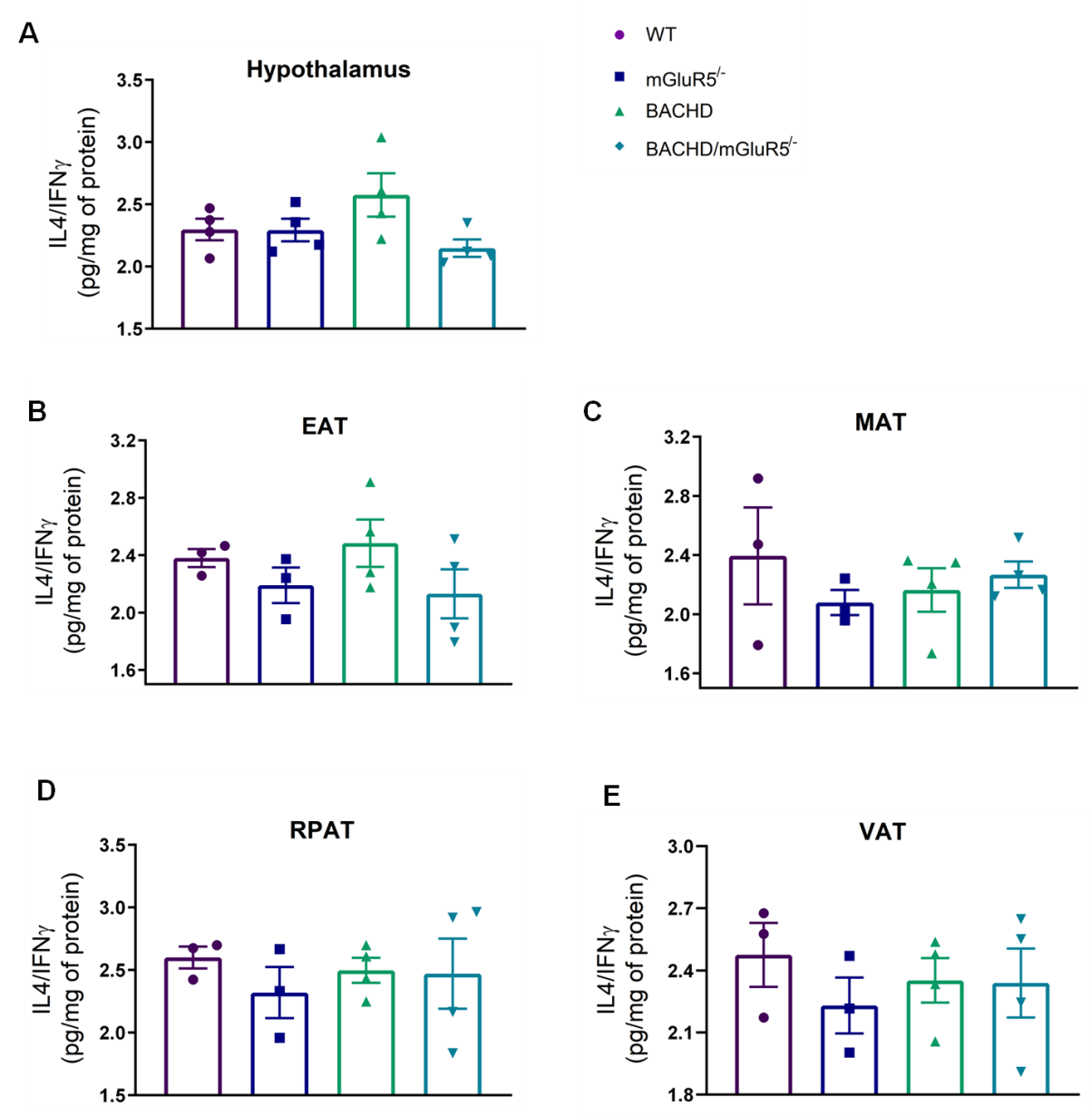


**Figure S8- At 6 months of age, IL-4/IFN ratio is not altered in the BACHD/mGluR5^-/-^ mice.** (**A**-**E**) IL-4/IFN ratio in the hypothalamus, epididymal (EAT), mesenteric (MAT), retroperitoneal (RPAT) adipose tissue, and in the sum of visceral adipose tissue (VAT) of WT, mGluR5, BACHD and BACHD/mGluR5^-/-^ mice, at 6 months of age. Error bars represent the mean ± SEM; n = 3-4. One-Way ANOVA followed by Bonferroni's post hoc test (**A**-**C,** and **E**), and One-Way Welch ANOVA followed by Games-Howell’s post hoc test (**D**).

**E**

**
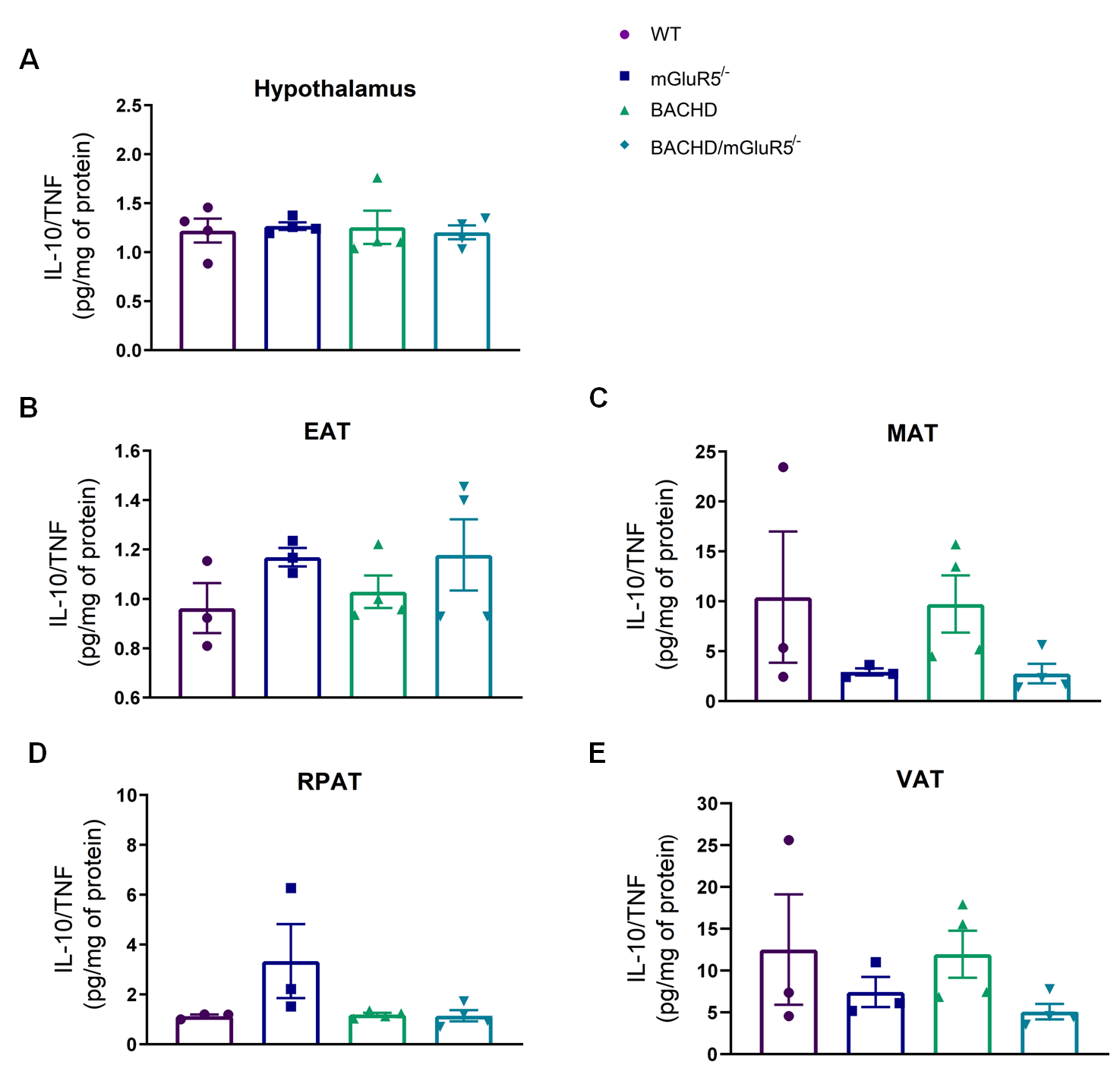
**

**Figure S9 – At 6 months of age, IL-10/TNF ratio is not altered in the BACHD/mGluR5^-/-^ mice.** (**A**-**E**) IL-10/TNF ratio in the hypothalamus, epididymal (EAT), mesenteric (MAT), retroperitoneal (RPAT) adipose tissue, and in the sum of adipose tissues (VAT) of WT, mGluR5, BACHD and BACHD/mGluR5^-/-^  mice, at 6 months of age. Error bars represent the mean ± SEM; n = 5-7. Krustal-Wallis followed by Dunn’s post hoc test (**A**), and One-Way Welch ANOVA followed by Games-Howell’s post hoc test (**B**-**E**).

**
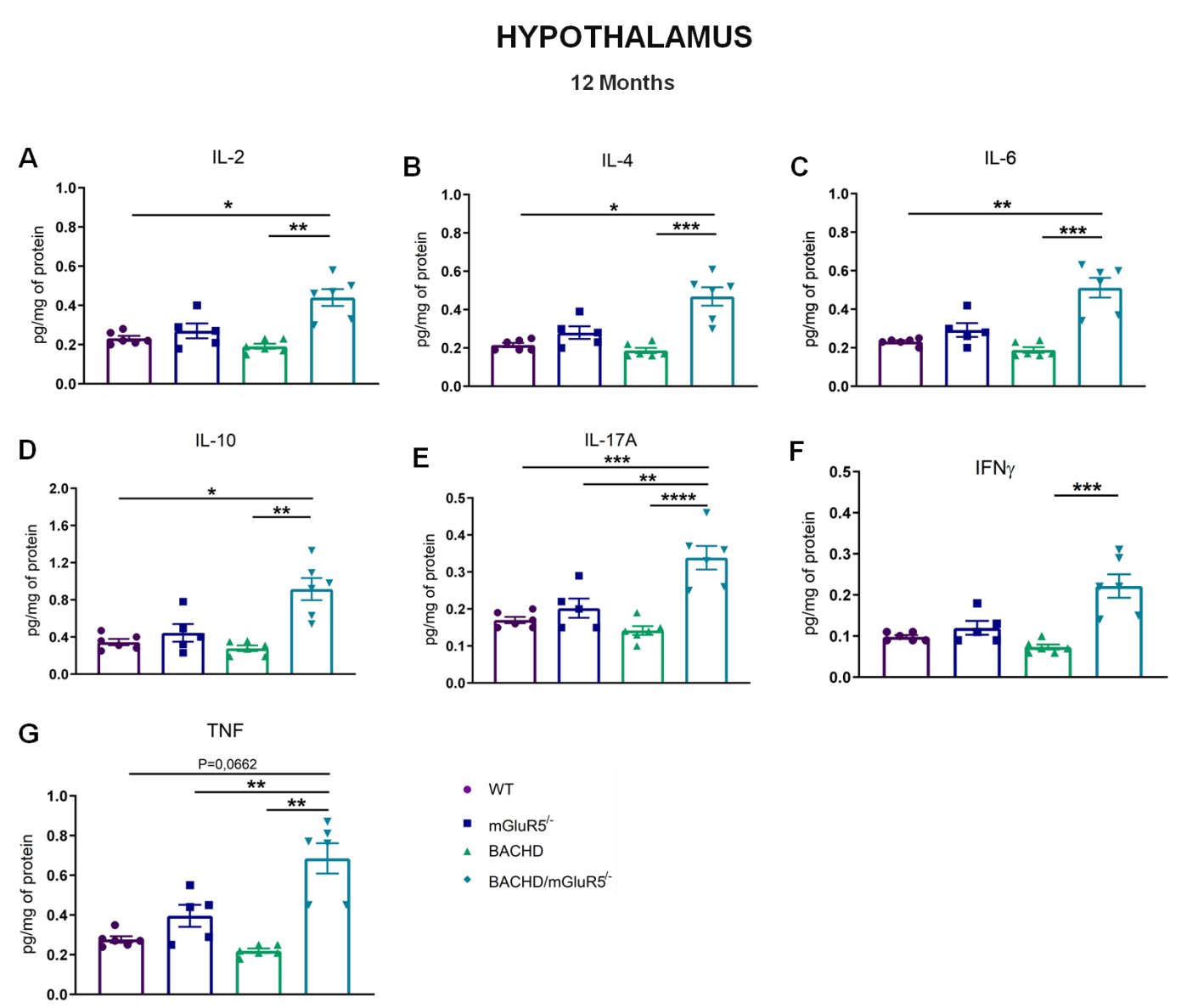
**

**Figure S10- At 12 months of age, BACHD/mGluR5^-/-^ mice presented an increase in the hypothalamic levels of pro-inflammatory and anti-inflammatory cytokines.** (**A**-**G**) Hypothalamic measurement, by cytometric bead assay (CBA), of the individual levels of IL-2, IL-4, IL-6, IL-10, IL-17A, INFγ, and TNF of WT, mGluR5, BACHD and BACHD/mGluR5^-/-^ mice, at 12 months of age. Error bars represent the mean ± SEM; n = 5-6. One-Way Welch ANOVA followed by Games-Howell’s post test (**A**, **D** and **G**), Krustal-Wallis followed by Dunn’s post hoc test (**B**, **C**, and **F**), and One-Way ANOVA followed by Bonferroni post hoc test (**E**). *P <0.05; **P <0.01; ***P <0.001; ****P <0.0001.


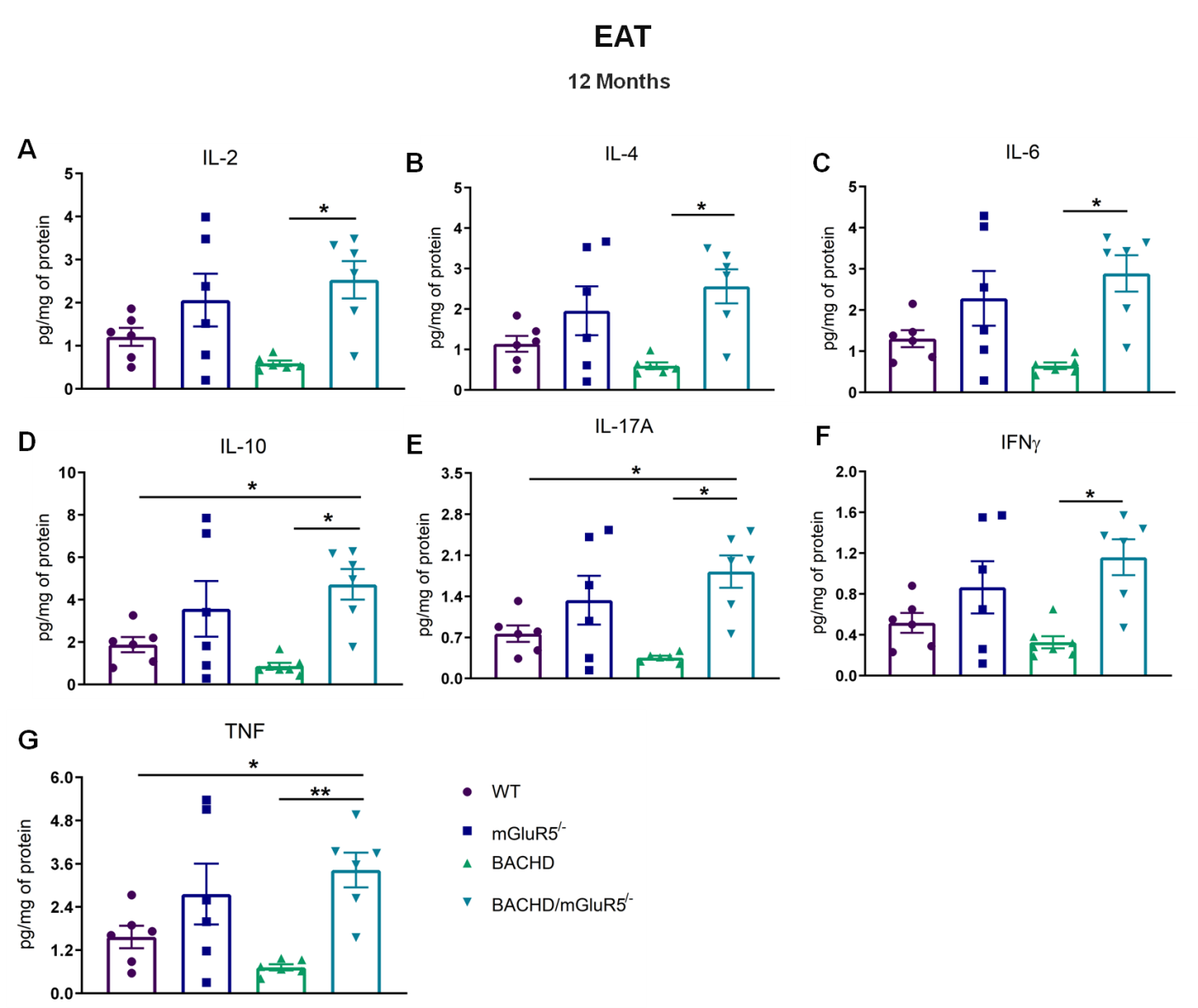


**Figure S11- BACHD/mGluR5^-/-^ mice presented an increase in pro-inflammatory and anti-inflammatory cytokines levels in epididymal adipose tissue (EAT).** (**A**-**G**) Measurement, by cytometric bead assay (CBA), of the individual levels of IL-2, IL-4, IL-6, IL-10, IL-17A, INFγ, and TNF in EAT of WT, mGluR5, BACHD and BACHD/mGluR5^-/-^ mice, at 12 months of age. Error bars represent the mean ± SEM; n = 5-6. One-Way Welch ANOVA followed by Games-Howell’s post hoc test. *P <0.05; **P <0.01.


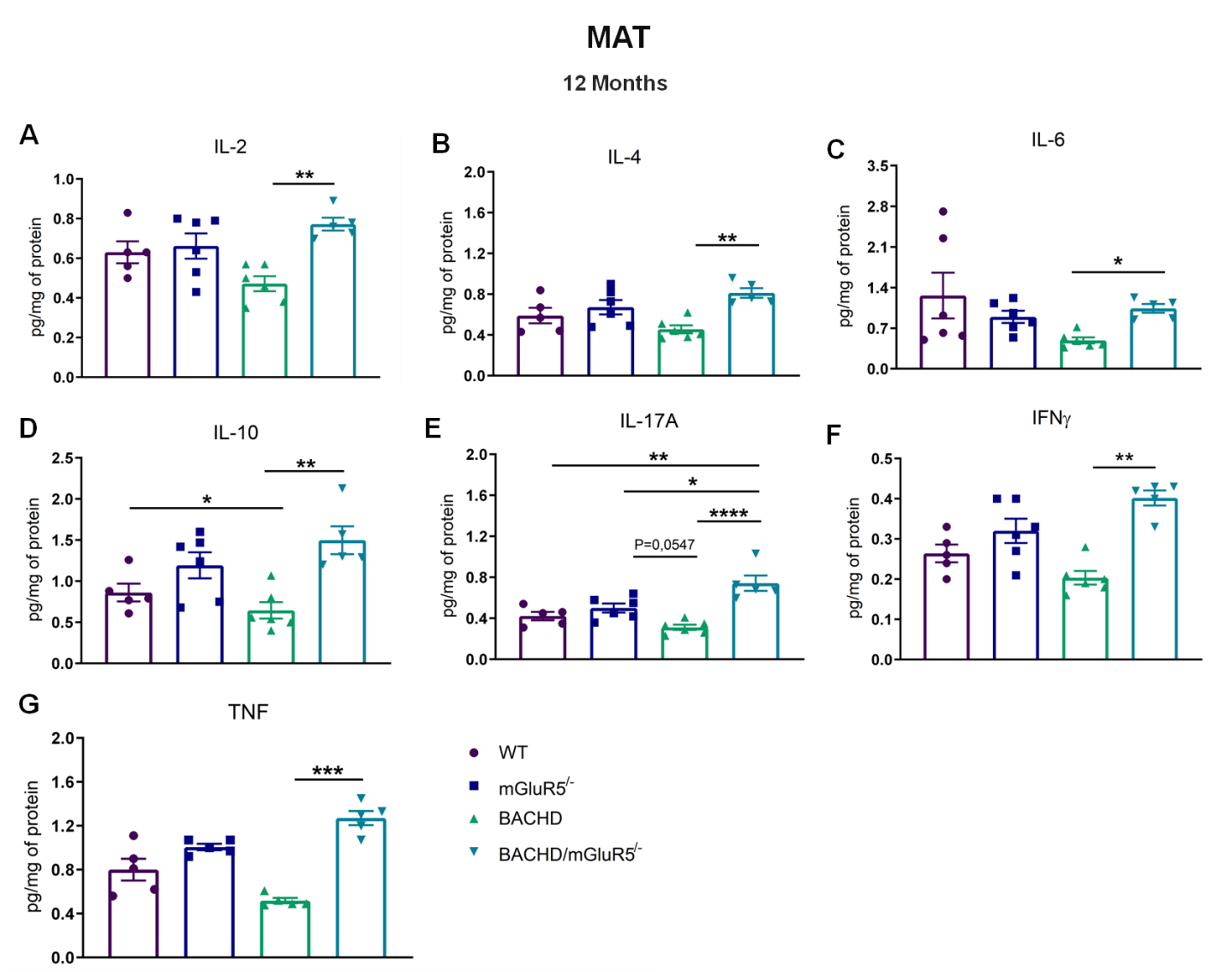


**Figure S12- BACHD/mGluR5^-/-^ mice presented an increase in pro-inflammatory and anti-inflammatory cytokines levels in mesenteric adipose tissue (MAT).** (**A**-**G**) Measurement, by cytometric bead assay (CBA), of the individual levels of IL-2, IL-4, IL-6, IL-10, IL-17A, INFγ, and TNF in MAT of WT, mGluR5, BACHD and BACHD/mGluR5^-/-^ mice, at 12 months of age. Error bars represent the mean ± SEM; n = 5-6. One-Way ANOVA followed by Bonferroni post hoc test (**A**, **B**, **D**, and **E**), and Krustal-Wallis followed by Dunn’s post hoc test (**C**, **F**, and **G**). *P <0.05; **P <0.01; ***P <0.001; ****P <0.0001.


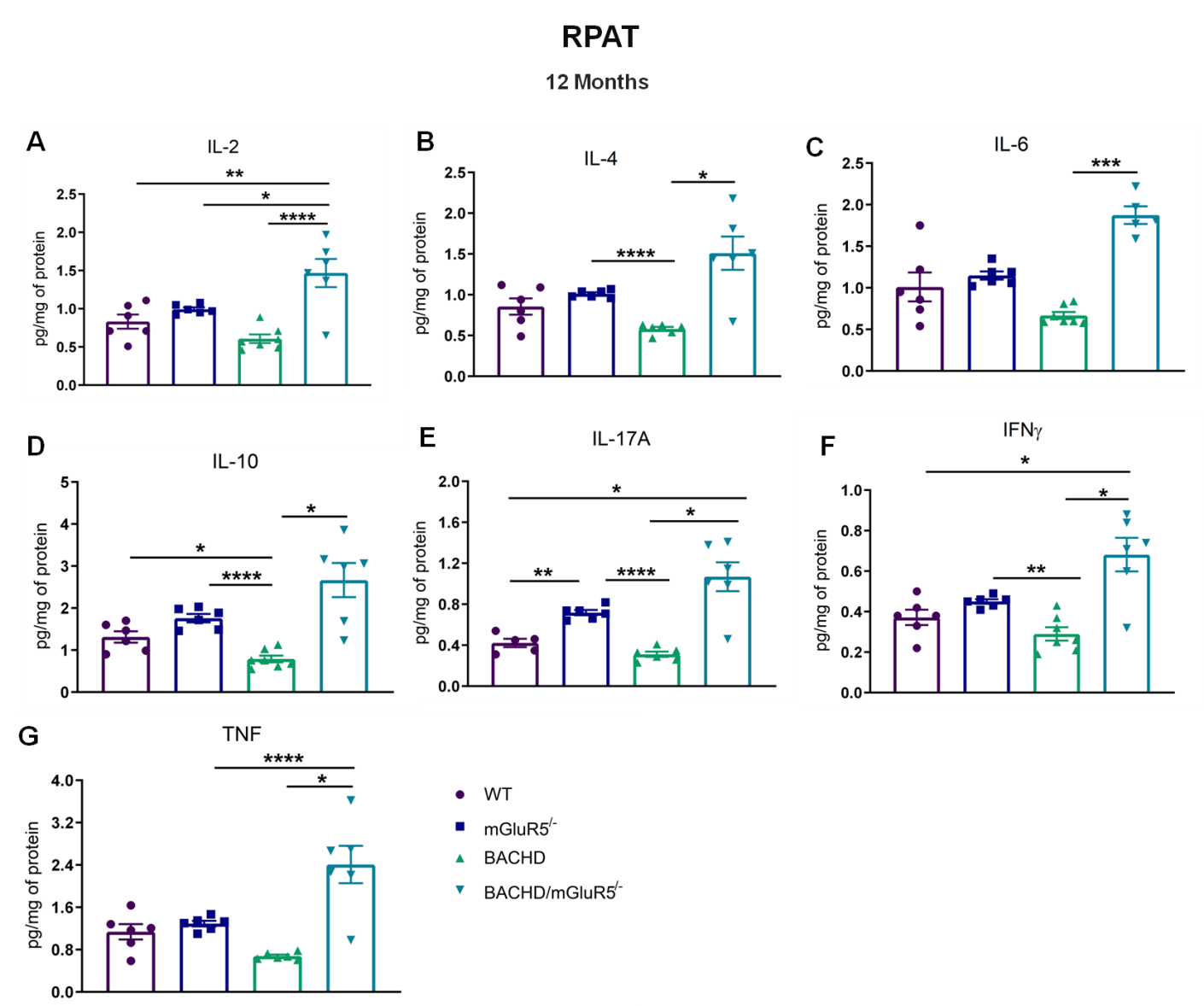


**Figure S13- BACHD/mGluR5^-/-^ mice presented an increase in pro-inflammatory and anti-inflammatory cytokines levels in retroperitonal adipose tissue (RPAT).** (**A**-**G**) Measurement, by cytometric bead assay (CBA), of the individual levels of IL-2, IL-4, IL-6, IL-10, IL-17A, INFγ, and TNF in RPAT of WT, mGluR5, BACHD and BACHD/mGluR5^-/-^ mice, at 12 months of age. Error bars represent the mean ± SEM; n = 5-6. One-Way ANOVA followed by Bonferroni post hoc test (**A**), and Krustal-Wallis followed by Dunn’s post test (**C**), and One-Way Welch ANOVA followed by Games-Howell’s post hoc test (**B**, **D**, **E**, **F**, and **G**). *P <0.05; **P <0.01; ***P <0.001; ****P <0.0001.
